# Ikaros mediates gene silencing in T cells through Polycomb repressive complex 2

**DOI:** 10.1038/ncomms9823

**Published:** 2015-11-09

**Authors:** Attila Oravecz, Apostol Apostolov, Katarzyna Polak, Bernard Jost, Stéphanie Le Gras, Susan Chan, Philippe Kastner

**Affiliations:** 1 Functional Genomics and Cancer, Institut de Génétique et de Biologie Moléculaire et Cellulaire (IGBMC), INSERM U964, CNRS UMR 7104, Université de Strasbourg, Equipe Labellisée Ligue Contre le Cancer, 1 rue Laurent Fries, Illkirch 67404, France; 2 IGBMC Microarray and Sequencing Platform, Illkirch 67404, France; 3 Faculté de Médecine, Université de Strasbourg, Strasbourg 67000, France

## Abstract

T-cell development is accompanied by epigenetic changes that ensure the silencing of stem cell-related genes and the activation of lymphocyte-specific programmes. How transcription factors influence these changes remains unclear. We show that the Ikaros transcription factor forms a complex with Polycomb repressive complex 2 (PRC2) in CD4^−^CD8^−^ thymocytes and allows its binding to more than 500 developmentally regulated loci, including those normally activated in haematopoietic stem cells and others induced by the Notch pathway. Loss of Ikaros in CD4^−^CD8^−^ cells leads to reduced histone H3 lysine 27 trimethylation and ectopic gene expression. Furthermore, Ikaros binding triggers PRC2 recruitment and Ikaros interacts with PRC2 independently of the nucleosome remodelling and deacetylation complex. Our results identify Ikaros as a fundamental regulator of PRC2 function in developing T cells.

## Abstract (web summary)

Haematopoietic stem and progenitor cell-specific genes are epigenetically silenced during T cell differentiation. Here the authors show that Ikaros represses over 500 loci in developing T cells in cooperation with PRC2 and independently of its well established partner NuRD.

## Graphical abstract

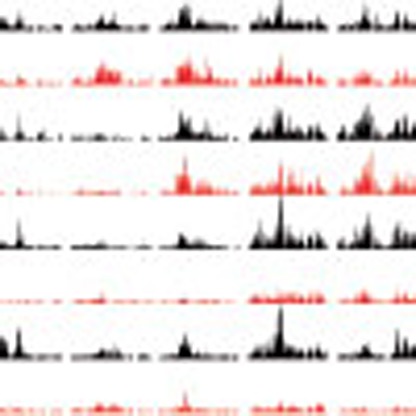

The development of a haematopoietic stem cell (HSC) into a T lymphocyte requires the loss of stem cell properties and the acquisition of T-cell characteristics, which is accompanied by changes in chromatin architecture and gene expression. Although genome-wide studies have begun to provide a detailed view of these changes and associated transcriptional regulators^1^,^2^,^3^, the current understanding is largely correlative and the impact of a given regulator in the dynamic evolution of the transcriptional and epigenetic states remains poorly understood.

The Ikaros transcription factor is critical for T-cell development. It is important early, for lymphoid specification in haematopoietic progenitors^4^, and late, to activate and repress numerous genes in thymocytes^5^,^6^. Ikaros shapes the timing and specificity of the Notch target gene response in double-negative (DN) CD4^−^CD8^−^ thymocytes^5^, and modulates positive and negative selection in double-positive (DP) CD4^+^CD8^+^ thymocytes^7^. Further, Ikaros is implicated in peripheral T-cell functions^8^,^9^,^10^,^11^. At the molecular level, Ikaros acts as both transcriptional repressor or activator. It associates with the nucleosome remodelling and deacetylation (NuRD) complex^12^,^13^, suggesting that it may repress transcription via NuRD-mediated histone deacetylation. In addition, it has been shown that Ikaros represses the expression of the Notch target gene *Hes1* in DP thymocytes^14^,^15^, which is correlated with decreased levels of histone H3 lysine 27 trimethylation (H3K27me3) in Ikaros-deficient cells, thus suggesting a possible role for Polycomb group proteins in Ikaros-dependent gene silencing. Collectively, these studies indicate that the molecular mechanisms of Ikaros-dependent repression remain unclear.

Here we show that loss of H3K27me3 is a prominent epigenetic defect in Ikaros-deficient thymocytes, which underlies the ectopic expression of genes repressed by Ikaros, including HSC-specific genes and Notch target genes. Ikaros is required for Polycomb repressive complex 2 (PRC2) binding to target loci in DN3 cells. Ikaros associates with PRC2 in DN cells and stable Ikaros–PRC2 complexes form independently of NuRD. Thus, Ikaros mediates gene silencing in T cells in large part through PRC2.

## Results

### Widespread loss of H3K27me3 in Ikaros-deficient DP cells

To assess the global effect of Ikaros on the ‘repressive' H3K27me3 and ‘active' histone H3 lysine 4 trimethyl (H3K4me3) marks, we compared DP thymocytes from 3- to 4-week-old wild-type (WT) and Ik^L/L^ mice by chromatin immunoprecipitation sequencing (ChIP-seq). Ik^L/L^ mice carry a hypomorphic mutation in the *Ikzf1* gene and the levels of functional Ikaros proteins in Ik^L/L^ cells are ∼10% of WT^14^,^16^. Although Ik^L/L^ mice die from T-cell acute lymphoblastic lymphomas/leukemias (ALL) at 4–6 months of age, the animals used here showed no signs of transformation in the thymus, as defined by CD4 and CD8 profiling, TCR Vα and Vβ chain usage, and the absence of intracellular Notch1 in DP thymocytes^14^,^15^.

**Figure 1:**
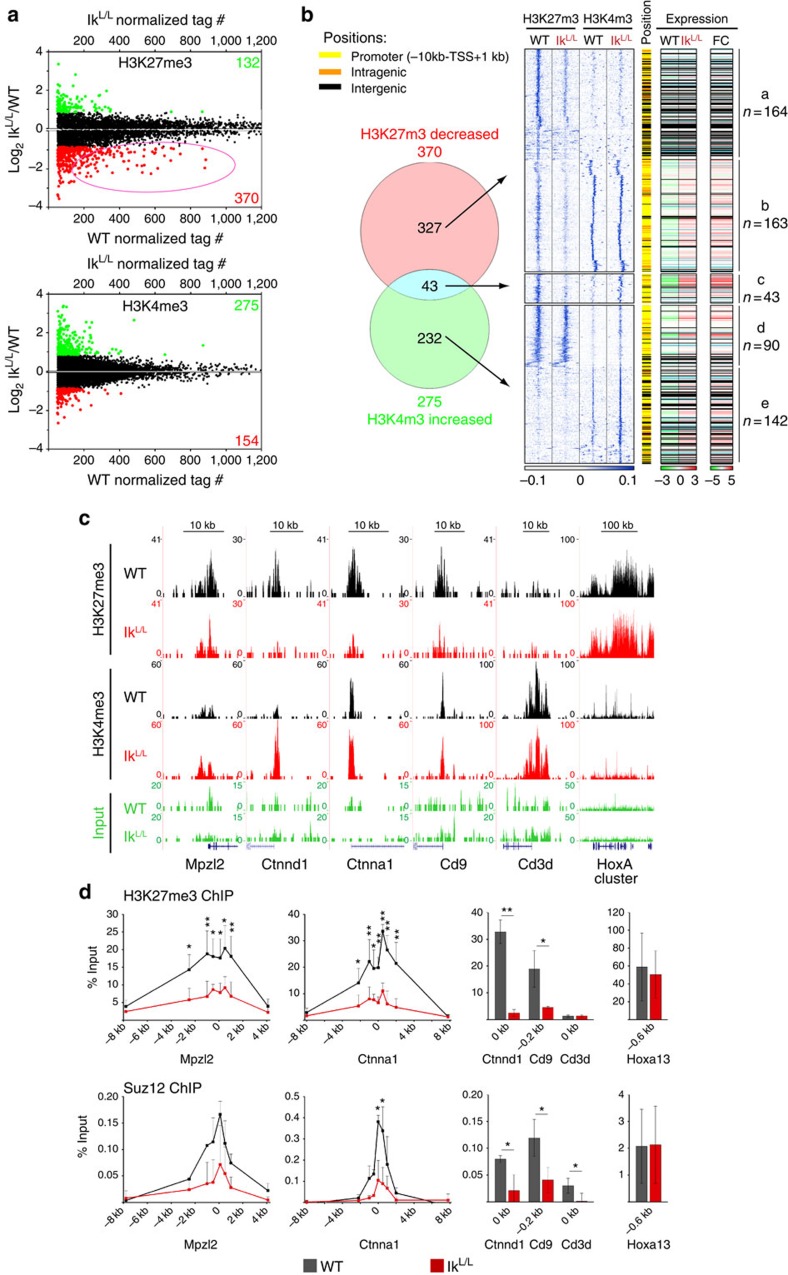
Impaired H3K27 trimethylation is a major defect in Ik^L/L^ DP thymocytes. (**a**) Scatter plots showing the Ik^L/L^/WT log_2_ fold changes of the indicated histone modifications in DP cells. ChIP-seq tag counts in WT and Ik^L/L^ are shown. Red/green values indicate the total number of chromatin marks that are >1.8 × decreased/increased in the mutant. Dots highlighted in the same colours represent the corresponding individual islands. The circled area highlights decreased regions with high tag counts in WT cells. (**b**) Venn diagram showing the overlap between genomic regions with decreased H3K27me3 or increased H3K4me3 in Ik^L/L^ DP thymocytes. The blue-coloured heatmap shows *k*-means clustering of the tag densities of the corresponding genomic regions for each group. The central stripe schematizes the genomic positions of the islands, with yellow, orange and black indicating promoter, intragenic and intergenic regions, respectively. The log_2_ expression (left) or log_2_ fold change (FC, right) of the matched genes is shown in the red–green heatmaps. Transcriptome data are from GSE 46090 (ref. 5). Black lines indicate intergenic regions and light blue lines indicate genes that were not represented on the microarray. For genes with multiple probe sets, we calculated a score corresponding to the product of the expression and FC values for each probe set, and selected the probe set with the highest score. (**c**) Representative UCSC Genome browser track of H3K27me3 and H3K4me3 ChIP-seq in WT and Ik^L/L^ cells. *Cd3d* and the HoxA cluster served as positive controls for the H3K4me3 and H3K27me3 tracks, respectively. (**d**) H3K27me3 and Suz12 ChIP–qPCRs from WT and Ik^L/L^ cells. The *x* axes indicate primer pair positions relative to the TSS of the test (*Mpzl2*, *Ctnna1*, *Ctnnd1* and *Cd9*) and control (*Cd3d* and *Hoxa13*) genes. % input=[(ab ChIP)-(IgG ChIP)]/1% input. Error bars, s.d.; *P<0.05, **P<0.01 (two-sample *t*-test); *n*=3–6 and *n*=2–3 for H3K27me3 and Suz12, respectively.

These experiments revealed 5,172 and 10,914 islands of enrichment for H3K27me3 and H3K4me3, respectively (Supplementary Fig. 1a). Although most were unchanged between WT and Ik^L/L^ cells (<1.8-fold), 370 of the H3K27me3 islands (7.2%) were decreased in Ik^L/L^ cells, many of which had high tag numbers in the WT sample (Fig. 1a). These islands could be divided into three major groups (Fig. 1b clusters *a*–*c*). Cluster *a* islands mapped mostly to intergenic regions and lacked H3K4me3 in both WT and Ik^L/L^ cells. Cluster *b* islands mapped largely to promoter or intragenic regions, and also exhibited H3K4me3 marks that were unchanged between WT and Ik^L/L^ cells (for example, *Ctnna1* and *Cd9*; Fig. 1c). By contrast, cluster *c* marked a small group of islands that showed a concomitant increase of H3K4me3 in the Ik^L/L^ sample (for example, *Mpzl2* and *Ctnnd1*; Fig. 1c). Furthermore, increased H3K4me3 was seen at 232 regions (2.2%) where H3K27me3 was either absent or unchanged (Fig. 1b clusters *d–e*). Finally, we found 132 regions with increased H3K27me3 and 154 regions with decreased H3K4me3, which overlapped only marginally (Fig. 1a and Supplementary Fig. 1b). Thus, a major hallmark of the chromatin landscape in Ik^L/L^ DP cells is the selective decrease of H3K27me3 at regions of ‘bivalent' H3K27me3/H3K4me3 chromatin (14.5% of the 1,382 bivalent regions).

We then assessed the impact of H3K27me3 and H3K4me3 defects on gene expression in Ik^L/L^ DP thymocytes, using our published microarray data (Fig. 1b and Supplementary Fig. 1b)^5^. Genes with decreased H3K27me3, and notably those with unaltered H3K4me3 (cluster *b*), were associated with increased messenger RNA expression in most cases (Fig. 1b right panels). Genes with a selective increase in H3K4me3 (Fig. 1b clusters *d–e*) were also associated with increased expression. In contrast, genes with increased H3K27me3, or decreased H3K4me3, were associated with slightly decreased mRNA levels (Supplementary Fig. 1b). These data indicate that diverse epigenetic changes underlie the deregulated gene expression in Ikaros-deficient DP cells. However, loss of H3K27me3 appears to be more prominent: it is the most frequent, occurs on islands with high H3K27me3 levels in WT cells and is often associated with increased gene expression regardless of H3K4me3 status. We therefore studied H3K27 trimethylation as a potential mechanism of Ikaros-mediated repression.

To determine whether the loss of H3K27me3 correlates with loss of PRC2 binding, we performed ChIP–quantitative PCR (qPCR) analysis to measure H3K27me3 levels and binding of the core PRC2 subunit Suz12 in WT and Ik^L/L^ DP thymocytes, using promoter-scanning primers (*Mpzl2* and *Ctnna1*) or primers specific for transcriptional start site (TSS) regions (*Cd9* and *Ctnnd1*; Fig. 1d). These experiments confirmed that the reduction of H3K27me3 is linked to decreased Suz12 binding in Ik^L/L^ cells at all genes tested.

### Impaired H3K27me3 and gene repression in Ik^L/L^ thymocytes

**Figure 2:**
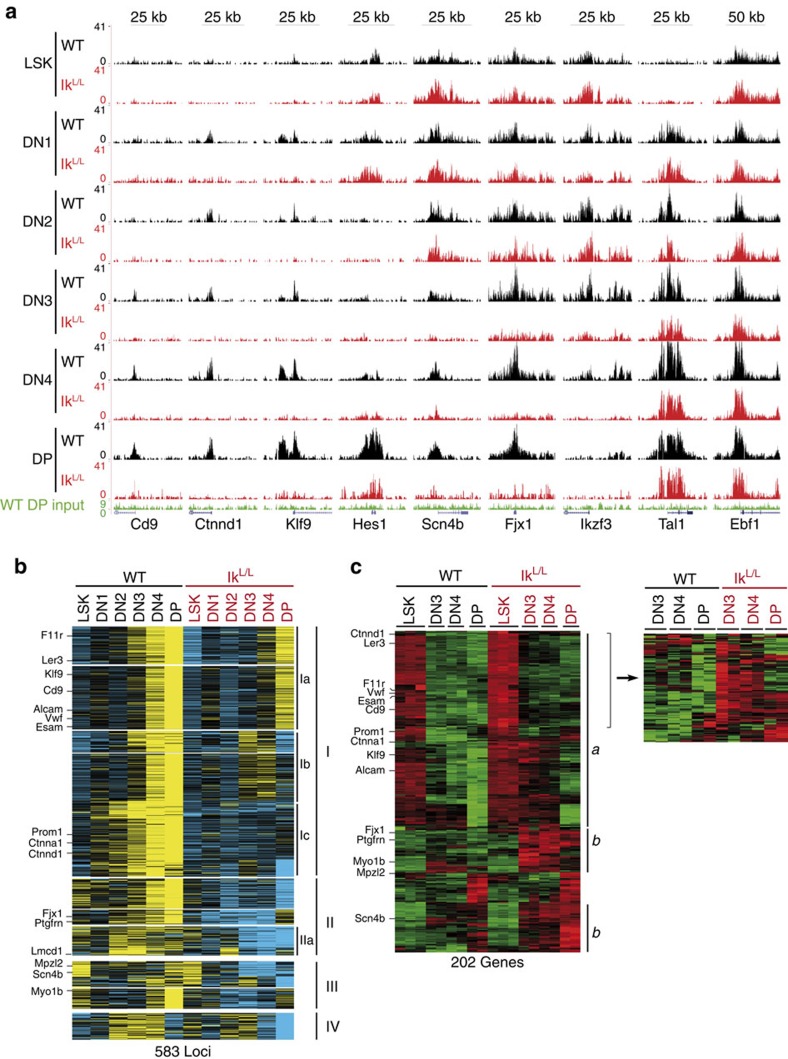
Ikaros is required for the establishment and maintenance of H3K27me3 in developing T cells. (**a**) Genome browser tracks of H3K27me3 ChIP-seq data from WT and Ik^L/L^ cells. (**b**) *k*-means clustering of relative normalized tag numbers (the region's normalized tag count/length of the region) in H3K27me3 enriched merged genomic regions from the indicated populations. Five hundred and eighty-three loci with >1.8 × decreased normalized tag count in at least one Ik^L/L^ population are shown. Blue and yellow represent low and high levels of H3K27me3, respectively. (**c**) *k*-means clustering of microarray data from the indicated populations showing 297 probe sets (202 genes) associated with decreased H3K27me3 (>1.8 × ) and an expression change of >4 × between the lowest and highest value of the analysed samples. The right panel shows clustering without the LSK data of part of cluster *a*. Green and red represent low and high levels of gene expression, respectively.

To evaluate the temporal changes in H3K27me3 during T-cell differentiation, we analysed the chromatin of bone marrow-derived LSK (Lin^−^Sca1^+^c-Kit^+^) cells and DN1 (CD44^+^CD25^−^), DN2 (CD44^+^CD25^+^), DN3 (CD44^−^CD25^+^), DN4 (CD44^−^CD25^−^) and DP thymocytes from 3- to 4-week-old WT and Ik^L/L^ mice. Overall, H3K27me3 levels were similar in Ik^L/L^ cells compared with WT (Supplementary Fig. 2a). By ChIP-seq, a total of 7,131 H3K27me3 islands were detected: the majority were similar between WT and Ik^L/L^ cells of the same developmental stage (Supplementary Fig. 2b) and included islands that were constitutively present (for example, *Ebf1*) or dynamically regulated (for example, *Tal1*; Fig. 2a). Of the islands that were changed, few were detected in the LSK and DN1 populations, whereas the number of islands with decreased H3K27me3 gradually increased from the DN2 to the DP stage (Supplementary Fig. 2c). Increased H3K27me3 was infrequent. In all, 583 islands were reduced >1.8-fold in the mutant samples compared with WT (Fig. 2b, Supplementary Fig. 2b and Supplementary Table 1); this number is likely to be underestimated, as some regions with a clear decrease in H3K27me3 were not selected by our bioinformatic criteria (for example, *Hes1* or *Ikzf3*; Fig. 2a). The reduced H3K27me3 islands could be further divided into four groups. Group I islands were small or undetectable in WT LSK cells, but increased from the DN1 to the DP stage (for example, *Cd9*, *Ctnnd1*, *Klf9*, *Ctnna1*, *Ier3*, *Vwf* and *Alcam*; Fig. 2a,b and Supplementary Fig. 2d); they were similar in Ik^L/L^ LSK cells, but did not increase (Fig. 2b subgroup Ic), or increased slightly in DP (subgroup Ia) or DN3 and DN4 (subgroup Ib) cells. Group II islands were constitutively present in WT LSK cells and thymocytes (for example, *Fjx1* and *Ptgfrn*); they were similarly observed in Ik^L/L^ LSK cells, but were undetectable in thymocytes after the DN2 and DN3 stages. Group III islands were present in LSK cells of both genotypes. In WT thymocytes, they were transiently reduced between the DN1 and DN3 stages, and increased in DN4 and DP cells. In Ik^L/L^ cells, these islands were also decreased from the DN1 stage, but did not increase afterwards, or increased only slightly in DP cells. Group III islands mapped to genes activated by Notch signalling (for example, *Mpzl2*, *Scn4b*, *Hes1* and *Myo1b* in Fig. 2a,b and Supplementary Fig. 2d)^5^ among others. Group IV islands were detected mainly between the DN2 and DN4 stages in WT cells; they were inconsistently detected in Ik^L/L^ LSK and DN cells, and were prematurely lost in DN4 cells (for example, *Ikzf3* and *Rorc* in Fig. 2a and Supplementary Fig. 2d).

To equate the H3K27me3 changes with gene expression in the above populations, we compared the mRNA expression of the associated genes between WT and Ik^L/L^ cells (LSK data from this study and thymocyte data from GSE 46090)^5^. Four hundred and forty-nine of the 583 H3K27me3 islands from Fig. 2b were associated with 444 genes, of which 392 were represented on the microarrays. Decreased H3K27me3 correlated with increased gene expression in each population by scatter plot analyses (Supplementary Fig. 3a). Indeed, 202 (52%) of the genes were dynamically regulated from the LSK to the DP stage, and 178 (88%) of these showed increased mRNA expression in the Ik^L/L^ cells compared with WT (Fig. 2c). Group *a* genes (*n*=114) were expressed in WT LSK cells and silenced during thymocyte differentiation (Supplementary Fig. 3b); these genes were also expressed in Ik^L/L^ LSK cells but were not silenced, or silenced less efficiently, in DN cells (group *a* and highlighted right panel in Fig. 2c). Group *a* included genes with important functions in HSC and progenitor cells (for example, *Cd9*, *Alcam*, *Ier3*, *F11r*, *Vwf*, *Esam* and *Prom1*)^17^,^18^,^19^,^20^,^21^,^22^,^23^ (Fig. 2a and Supplementary Fig. 2d for selected H3K27me3 profiles). Most group *a* genes (*n*=105) were associated with group I H3K27me3 islands (similarly represented among subgroups I, a–c in Fig. 2b). Group *b* genes (*n*=64) were not expressed in WT and Ik^L/L^ LSK cells, or in WT thymocytes, but were ectopically expressed in Ik^L/L^ thymocytes. These genes were associated in similar numbers with H3K27me3 islands from groups I (*n*=24), II (*n*=20) and III (*n*=20). Certain Notch-activated genes ectopically increased at the mRNA level in Ik^L/L^ thymocytes were found in group *b* (for example, *Myo1b*, *Lmcd1* and *Fjx1*)^5^.

Collectively, these analyses indicate that Ikaros deficiency results in the reduction or disappearance of >500 H3K27me3 islands during thymocyte development, and that this is correlated with ectopic expression of genes normally expressed in HSCs.

### Ikaros acts in DN cells to establish and maintain H3K27me3

To address the role of Ikaros in H3K27me3 regulation, we first studied Ikaros binding in WT DP cells by ChIP-seq. Unexpectedly, little or no Ikaros binding was detected at loci where H3K27me3 was reduced in Ik^L/L^ DP cells, although Ikaros bound to other places in the genome (Supplementary Fig. 4a). Given that H3K27me3 changes were also detected in Ik^L/L^ DN cells, we hypothesized that Ikaros influenced H3K27 trimethylation at earlier stages. Indeed, Ikaros proteins were abundant in DN2–DN4 cells (Supplementary Fig. 4b).

**Figure 3:**
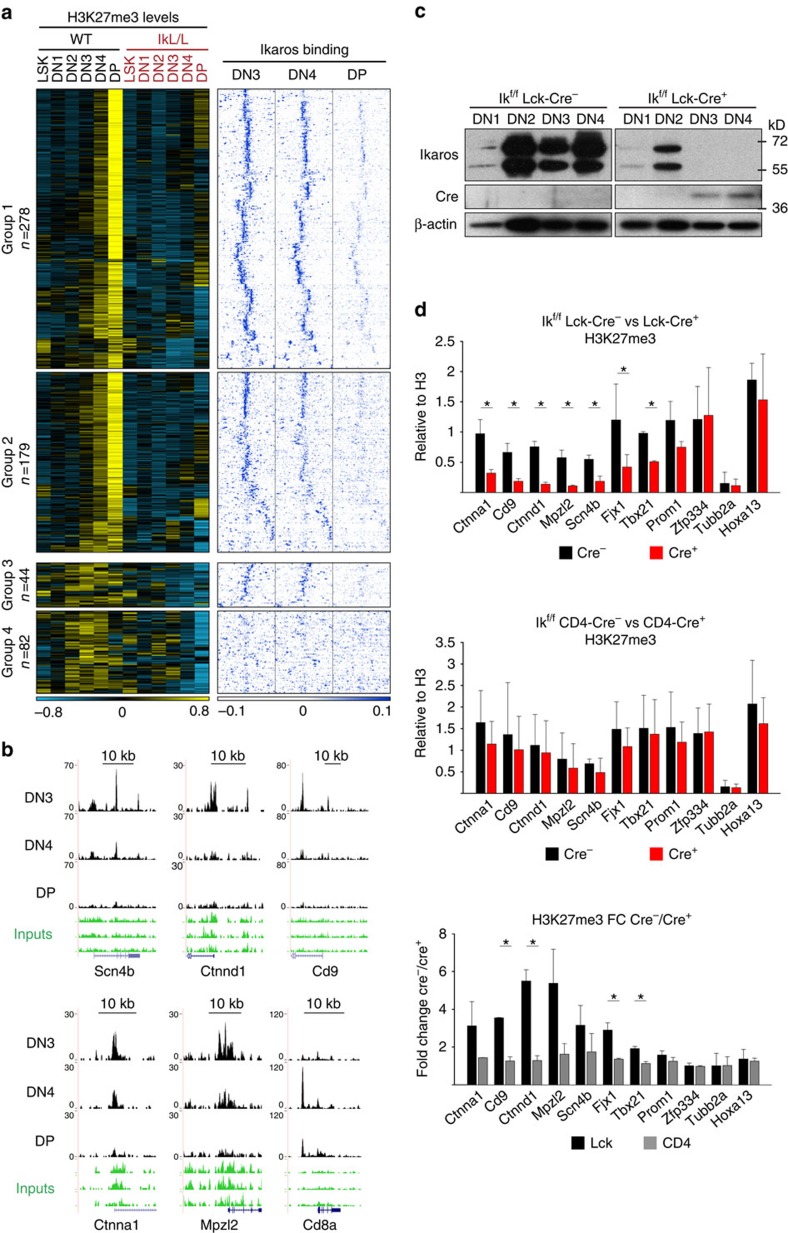
Ikaros binds to target genes and regulates H3K27me3 in DN3 cells. (**a**) Comparison of H3K27me3 profiles and Ikaros binding. The 583 H3K27me3 islands from Fig. 2 were first divided into those with increasing H3K27me3 from the DN3 to DP stage (groups 1 and 2) or not (groups 3 and 4). They were then further divided into groups with significant Ikaros binding (*P*≤10^−7^; groups 1 and 3) or not (groups 2 and 4). The H3K27me3 profiles of each group were clustered (*k*-means, left panels). Tag density heatmaps of the Ikaros ChIP-seq data centred around the corresponding H3K27me3 islands (±10 kb) were calculated for each group and clustered (*k*-means; right panels). (**b**) Representative genome browser tracks showing Ikaros binding. (**c**) Deletion of Ikaros in DN3 cells. Immunoblots of whole-cell lysates from DN1 (2 × 10^4^) and DN2–4 (5 × 10^4^) cells from Ik^f/f^ Lck-Cre^−^ and Ik^f/f^ Lck-Cre^+^ mice. The two bands detected with the anti-Ikaros antibody represent the predominant Ik1 and Ik2 isoforms. Representative of three independent experiments. (**d**) Ikaros deletion in DN3, but not DP, cells results in the loss of H3K27me3 at Ikaros target genes. Hoxa13 and Tubb2a are positive and negative controls, respectively. H3K27me3 ChIP–qPCRs on chromatin from DP cells of Ik^f/f^ Lck-Cre^−^ versus Ik^f/f^ Lck-Cre^+^ (*n*=2), Ik^f/f^ CD4-Cre^−^ versus Ik^f/f^ CD4-Cre^+^ (*n*=2) mice. The values indicate [(H3K27me3 ChIP)-(IgG ChIP)]/[(H3 ChIP)-(IgG ChIP)]. Cre^−^ over Cre^+^ fold changes are shown at the bottom. Error bars, s.d.; *P<0.05 (two-sample *t*-test).

Ikaros binding was studied in WT DN3, DN4 and DP cells by ChIP-seq (DN1 and DN2 cells were not analysed due to their low numbers). We found 37,050 Ikaros peaks in DN3, 23,656 in DN4 and only 5,351 in DP cells. The majority of the regions bound by Ikaros in DP cells (4,689, 87.6%) were also bound in DN3 and DN4 cells, and only a small number (132, 2.5%) were specific to DP cells, suggesting that Ikaros binds most of its target genes at the DN stage (Supplementary Fig. 4c). Interestingly, the proportion of promoter-associated peaks increased, whereas those at intergenic regions or gene bodies decreased, during differentiation (Supplementary Fig. 4d). The AGGAA motif was highly enriched among Ikaros peaks (Supplementary Fig. 4e), consistent with previous results^5^,^6^,^24^,^25^,^26^. Ikaros peaks were also enriched for Ctcf sites, as described^6^. Additional enriched motifs included E-boxes as well as those specific for Sp1, Runx1, Ets1, Nrf1, Zbtb33, Bhlhb2 and Nfya. We then compared the H3K27me3 profiles with Ikaros binding and found that the majority of the H3K27me3 islands (322/583) reduced in Ik^L/L^ thymocytes (Fig. 2b) overlapped with Ikaros binding in WT cells (331 peaks, *P*≤10^−7^; Fig. 3a groups 1 and 3, and Fig. 3b). Ikaros binding was most pronounced in DN3 and DN4 cells, and was faint or absent in DP cells. An additional 179 H3K27me3 islands showed clear Ikaros enrichment even though they were not identified bioinformatically (group 2). In contrast, little Ikaros binding was detected at regions where the H3K27me3 profiles were more dispersed (group 4 in Fig. 3a, which corresponded mostly to groups IIa and IV in Fig. 2b). Thus, nearly all of the H3K27me3 islands that increased with differentiation in WT cells show concomitant Ikaros binding in DN3 and DN4 cells. However, there was no correlation between the size of Ikaros peaks and the magnitude of H3K27me3 changes (Supplementary Fig. 4f).

To determine whether Ikaros is required in DN thymocytes for H3K27 trimethylation at these genes, we studied two T-cell-specific conditional Ikaros-null mouse models: Ik^f/f^ Lck-Cre^+^ mice, in which Ikaros proteins were undetectable from the DN3 stage (Fig. 3c and Supplementary Fig. 5a), and Ik^f/f^ CD4-Cre^+^ mice, in which Ikaros expression was lost from the DP stage^5^. As these mice developed T-ALL with similar kinetics as Ik^L/L^ animals (Supplementary Fig. 5b–d)^5^, DP cells were purified from 3- to 4-week-old mice and studied for H3K27me3 at select loci by ChIP–qPCR analysis (Fig. 3d). We analysed *Ctnna1*, *Cd9* and *Ctnnd1*, because these loci gradually acquire H3K27me3 from the DN1 stage on in WT but not Ik^L/L^ cells, and *Mpzl2*, *Scn4b*, *Fjx1*, *Tbx21*, *Prom1* and *Zfp334*, because they are constitutively marked with H3K27me3 in all WT populations, but lose this mark in Ik^L/L^ DN cells at various stages (Fig. 2a and Supplementary Fig. 2d). We found that H3K27me3 levels were similar to WT at all genes in Ik^f/f^ CD4-Cre^+^ DP cells, but were lower in Ik^f/f^ Lck-Cre^+^ DP cells, except for *Prom1* and *Zpf334*. Interestingly, *Zfp334* and *Prom1* lost H3K27me3 early during differentiation in Ik^L/L^ cells (in DN1 and DN2 cells, respectively; Supplementary Fig. 2d). These results demonstrate that Ikaros acts in DN cells in a stage- and locus-specific manner, to initiate and/or maintain H3K27me3.

### Reduced PRC2 binding to specific loci in Ik^L/L^ DN3 cells

To determine the impact of Ikaros on PRC2 recruitment, we first evaluated Ezh2 protein levels in thymocytes. Ezh2 was detected in both WT and Ik^L/L^ DN subsets (Supplementary Fig. 6a), indicating that the decrease in H3K27me3 in Ikaros-deficient thymocytes is not due to a decrease in PRC2 activity.

**Figure 4:**
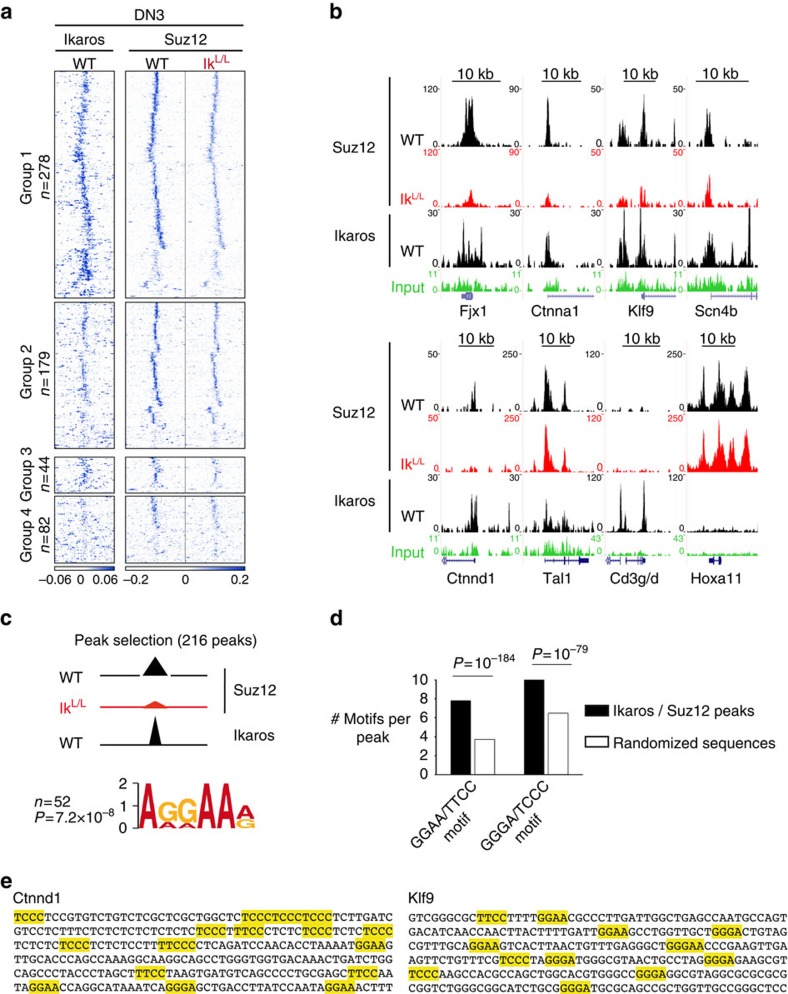
Ikaros is required for PRC2 targeting in DN3 cells. (**a**) *k*-means clustering of Ikaros and Suz12 tag density heatmaps on the 583 regions that showed decreased H3K27me3 in Ik^L/L^ DN3 cells. Groups 1–4 are identical to those in Fig. 3a. (**b**) Representative genome browser tracks showing Suz12 and Ikaros ChIP-seq in WT and Ik^L/L^ cells. WT DP input controls in green. (**c**) The Ikaros peaks associated with the 216 Suz12 peaks (from Supplementary Fig. 5b left) were selected and motif enrichment near the peak centre (±40 bp from the summit) was analysed with MEME. The AGGAA motif was significantly enriched. (**d**) Enrichment of the GGGA and GGAA motifs near the Ikaros/Suz12 peaks. The average number of GGGA and GGAA motifs (or their complementary motifs) were determined within 150 bp of the peak summits defined in Supplementary Fig. 5b, as well as in nucleotide sequences of randomized permutations. The *P*-value was calculated with the *χ*^2^-test. (**e**) Examples of sequences where the putative Ikaros target motifs GGGA and GGAA have been highlighted.

We then evaluated Suz12 binding by ChIP-seq in DN3 cells, where Ikaros binding was best detected, and identified 9,541 Suz12 peaks (*P*≤10^−7^) in the WT and Ik^L/L^ samples. Although most Suz12 peaks were comparable between WT and mutant, the majority of the 583 loci, characterized by reduced H3K27me3 in Ik^L/L^ cells, showed a striking overlap between Ikaros and Suz12 binding on WT chromatin and a marked decrease in Suz12 enrichment on Ik^L/L^ chromatin (Fig. 4a,b). Of these, we focused on 216 Suz12 peaks that overlapped with Ikaros binding using stringent bioinformatic criteria (*P*≤10^−7^; Supplementary Fig. 6b left). The genes associated with these peaks exhibited a clear bias towards activation in Ik^L/L^ DN3 cells (Supplementary Fig. 6c), compared with other groups. The Ikaros motif (AGGAAa/g) was the only known motif detected under the Ikaros/Suz12 peaks and it was located within 40 bp of the centre of the Ikaros peaks (Fig. 4c). The regions surrounding the Ikaros peaks (±150 bp of the summit) also contained a high number of GGAA or GGGA motifs (Fig. 4d,e), previously shown to bind Ikaros^27^,^28^,^29^, when compared with control sequences with random nucleotide permutations, suggesting a functional importance. Thus, Ikaros is required for PRC2 binding to a specific set of target genes in DN3 cells.

### Ikaros is required for PRC2 binding and H3K27 trimethylation

To determine how Ikaros induces PRC2 binding, we established a gain-of-function system with the ILC87 Ikaros-null T-cell line, derived from a lymphoma of an Ik^f/f^ Lck-Cre^+^ mouse (Supplementary Fig. 7a). ILC87 cells were stably transduced to express an inducible full-length Ikaros1 isoform fused to the ligand-binding domain of the oestrogen receptor (Ik1-ER) and green fluorescent protein (GFP). Upon treatment with the ER ligand 4-hydroxytamoxifen (4OHT), for 1–3 days, GFP^+^ cells showed enhanced nuclear translocation of Ik1-ER (Supplementary Fig. 7b)^5^. The differentiation markers CD4, CD8 and CD3 showed little change in 4OHT-treated ILC87-Ik1-ER cells after 1 day (Supplementary Fig. 7c), although their expression increased at later timepoints.

**Figure 5:**
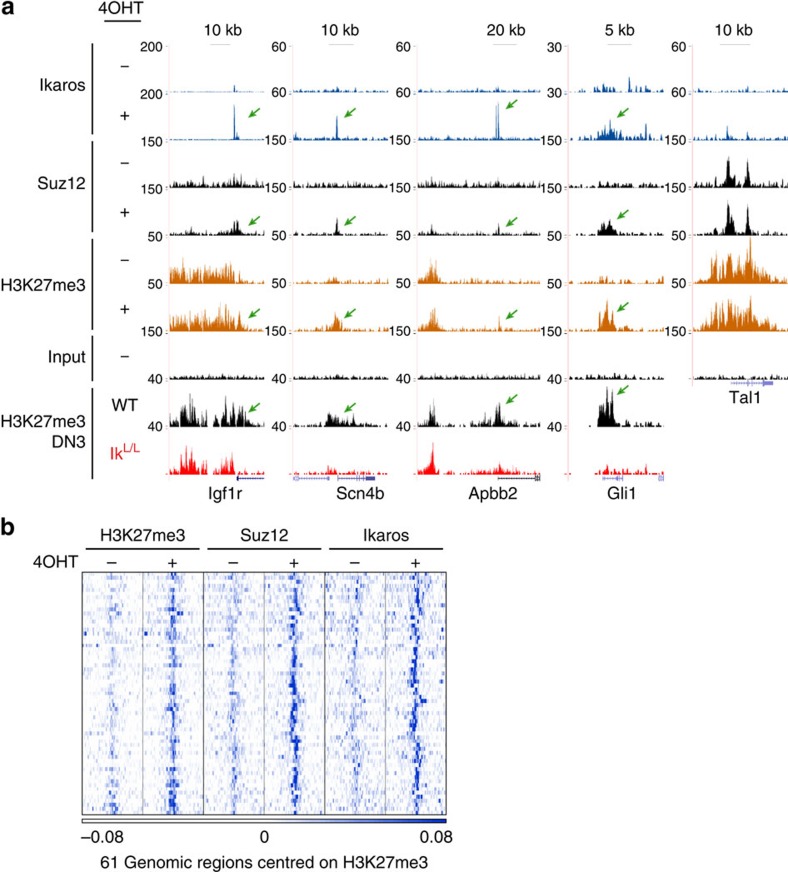
Ikaros induces PRC2 targeting and H3K27 trimethylation. ChIP-seq of Ikaros, Suz12 and H3K27me3 on ILC87-Ik1-ER cells treated with 4OHT (+) or ethanol (−) for 1 day (Ikaros) or 3 days (Suz12 and H3K27me3). (**a**) Representative genome browser tracks. Green arrows depict induced Ikaros, Suz12 and H3K27me3. Corresponding H3K27me3 tracks from primary WT and Ik^L/L^ DN3 thymocytes are shown at the bottom. Tal1 is shown as positive control. Vertical scales indicate tag numbers. (**b**) *k*-means clustering of H3K27me3-centred (±10 kb) Ikaros, Suz12 and H3K27me3 tag density heatmaps on the 61 regions with increased H3K27me3 in 4OHT-treated samples. Selected regions were identified bioinformatically (as having an Ikaros peak in 4OHT-treated cells, which overlapped with a H3K27me3 island that increased >1.8 × between ethanol- and 4OHT-treated cells), or by visual scanning of the tracks.

We analysed the genomic localization of Ikaros, Suz12 and H3K27me3 in 4OHT-treated ILC87-Ik1-ER cells by ChIP-seq. Ikaros was studied after 24 h of treatment, and Suz12 and H3K27me3 after 72 h. Although Ikaros binding was nearly absent in vehicle-treated cells, 4OHT induced binding to 8,017 regions (Supplementary Fig. 8a). We identified 61 sites with increased H3K27me3 that correlated with a 4OHT-dependent increase of Ikaros and/or Suz12 binding (Fig. 5, Supplementary Fig. 8b and Supplementary Table 2). Importantly, 40 of these sites mapped to genes that showed decreased H3K27me3 in primary Ikaros-deficient thymocytes (Fig. 5a, Supplementary Fig. 8b and Supplementary Table 2). Thus, gain of Ikaros function in ILC87 cells rescues H3K27me3 at genes that had lost the mark in Ikaros-deficient thymocytes. These data were validated by ChIP–qPCR analysis at the *Scn4b* locus (Supplementary Fig. 8c). Similar results were obtained with ILC87 cells transduced to constitutively express non-tagged Ikaros (Supplementary Fig. 8d).

**Figure 6:**
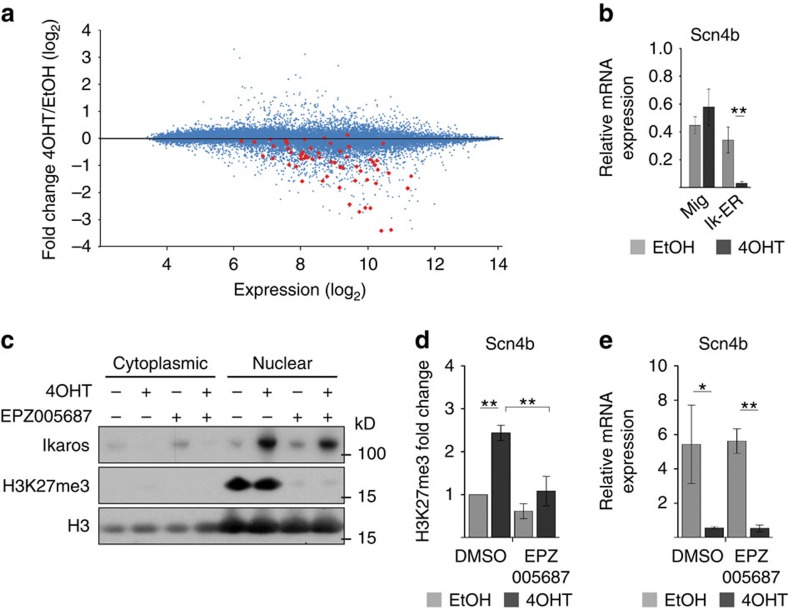
Impact of Ikaros-induced PRC2 activity on gene expression. (**a**) MA plot depicting the expression (in ethanol-treated samples) versus fold change of all the probe sets of the Mouse Gene ST 1.0 array in ILC87-Ik1-ER cells treated with ethanol or 4OHT for 1 day. Probe sets highlighted in red represent the 61 genes with 4OHT-induced H3K27me3 from Fig. 5b. (**b**) Reverse transcriptase–qPCR (RT–qPCR) analysis of *Scn4b* expression in ILC87-Ik1-ER and control (Mig) cells treated with ethanol or 4OHT for 1 day. (**c**) Western blotting of H3K27me3 and Ikaros in ILC87-Ik1-ER cells treated with the Ezh2 inhibitor EPZ005687 or vehicle (dimethyl sulphoxide (DMSO)) for 2 days. During day 2, cells were also treated with 4OHT or ethanol. Histone H3 is shown as loading control. (**d**,**e**) H3K27me3 ChIP–qPCR (**d**) and RT–qPCR (**e**) analysis of the *Scn4b* gene in cells treated as in **c**. Expression data are normalized to Hprt. Error bars, s.d. (*n*=3); *P<0.05, **P<0.01 (two-sample *t*-test).

To determine whether increased H3K27me3 correlates with decreased gene expression, we generated microarray data from ILC87-Ik1-ER cells treated with 4OHT or vehicle and evaluated the mRNA level of the 61 genes that exhibited increased H3K27me3. Most of the genes showed reduced mRNA expression (Fig. 6a), which was confirmed by reverse transcriptase–qPCR analysis for *Scn4b* (Fig. 6b), suggesting a correlation between H3K27me3 and gene repression. To determine whether increased H3K27me3 is required, we analysed 4OHT-treated ILC87-Ik1-ER cells treated with an Ezh2 inhibitor. This strongly reduced both the global H3K27me3 levels and the 4OHT-induced H3K27me3 increase at *Scn4b* (Fig. 6c,d), but *Scn4b* expression was still repressed (Fig. 6e). Thus, Ikaros is associated with PRC2 binding and H3K27me3, but these events are not required to initiate Ikaros-mediated repression of this gene.

### Ikaros interacts with PRC2 independently of NuRD

**Figure 7:**
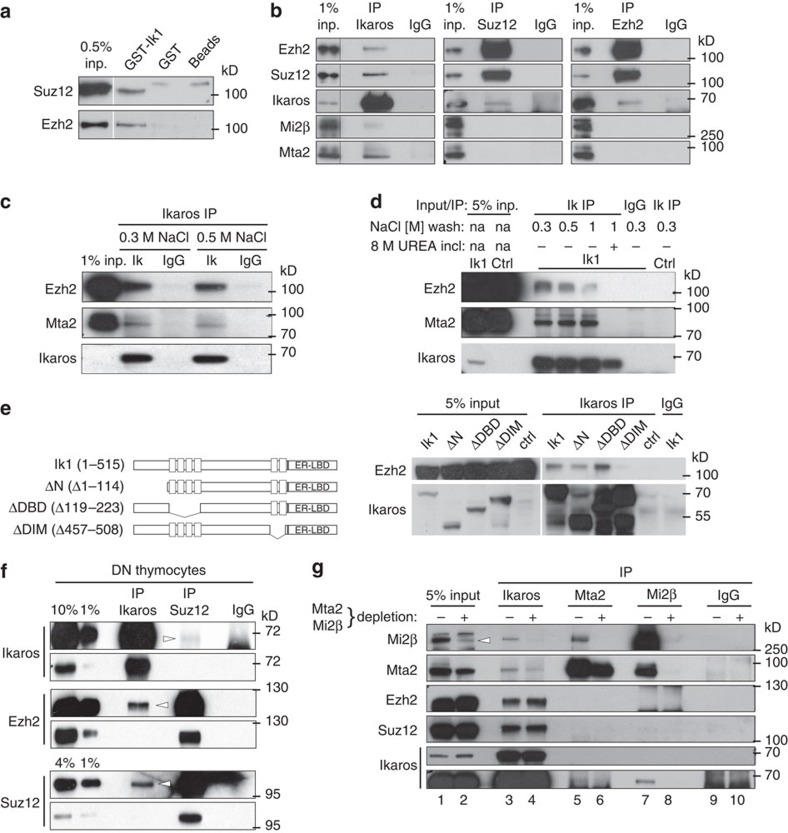
Ikaros forms a complex with PRC2 independent of NuRD. (**a**) GST-Ikaros binds PRC2. Bacterially expressed Ik1-GST fusion protein was immobilized on glutathione-agarose beads and incubated with ILC87 nuclear extracts. Bound proteins were analysed by western blotting. Immobilized GST protein alone, or glutathione-agarose beads alone were used as negative controls. (**b**) Ikaros interacts with PRC2 in ILC87 cells. Western blottings for the indicated proteins after IP of Ikaros, Suz12, Ezh2 or IgG. One per cent inputs from the nuclear extracts of ILC87-Ik1-Bcl2 cells are shown. (**c**) Western blottings for the indicated proteins after IP of Ikaros (Ik) or IgG from nuclear extracts as in **b**, except that the IPs and washing steps were performed in the presence of 0.3 or 0.5 M NaCl as indicated. (**d**) Ikaros (Ik) or IgG IPs from nuclear extracts as in **b**. Immune complexes were washed in the presence of 0.3, 0.5 or 1 M NaCl, or 1 M NaCl plus 8 M urea, as indicated. The ctrl sample is an Ikaros IP performed on nuclear extracts of ILC87-Bcl2 cells transduced with the empty MigR1 vector. Five per cent inputs are shown. (**e**) Right: analysis of the interaction of Ikaros deletion mutants with Ezh2 by co-IP. As in **b**, except that ILC87-Bcl2 cells expressing the indicated Ikaros mutants were analysed. Five per cent input controls are shown. Left: schematic representation of the Ikaros deletion constructs. (**f**) Ikaros interacts with PRC2 in primary WT DN cells. Western blottings for the indicated proteins after IP of Ikaros, Suz12 or IgG on ∼333 μg nuclear extracts from WT CD4^−^CD8^−^CD3^−^ thymocytes. (**g**) NuRD depletion does not affect the Ikaros–PRC2 interaction. Nuclear extracts of ILC87-Ik1-Bcl2 cells were incubated in three consecutive steps with IgG- (−), or anti-Mta2- and anti-Mi2β-coupled (+), Protein A/G Sepharose beads. Resulting supernatants (shown as 5% inputs) were subjected to Ikaros, Mta2, Mi2β or IgG IPs and immunoblotted as indicated. Arrowhead indicates the Mi2β-specific signal. Short and long exposures of the Ikaros blot are shown. (**b**) Representative of ≥5, (**c**) 1, (**d**) 2, (**a**,**e**–**g**) 3 independent experiments.

The above results suggested that Ikaros may recruit PRC2 to its target genes. To determine whether Ikaros forms a complex with PRC2, we performed a glutathione *S*-transferase (GST) pull-down assay. Ikaros–GST fusion proteins were captured with glutathione beads and were incubated with nuclear extracts from ILC87 cells. Ikaros–GST, but not GST or empty glutathione beads, precipitated the PRC2 components Ezh2 and Suz12 (Fig. 7a). Furthermore, immunoprecipitation (IP) of nuclear extracts from ILC87 cells constitutively expressing Ikaros, with an anti-Ikaros antibody, led to the co-IP of Ezh2 and Suz12 (Fig. 7b). Conversely, IP of either Suz12 or Ezh2 led to the co-IP of Ikaros. As a positive control, the NuRD complex proteins Mi2β and Mta2 were also found to co-IP with Ikaros. Importantly, Mi2β and Mta2 did not co-IP with Suz12 or Ezh2, indicating that the interaction between PRC2 and Ikaros is specific. To assess the stability of the Ikaros–PRC2 interaction, the extracts were immunoprecipitated with the anti-Ikaros antibody in the presence of high salt concentrations (Fig. 7c); this showed that Ikaros could associate with Ezh2 and Mta2 in the presence of 0.3 or 0.5 M NaCl. In a second experiment, the extracts immunoprecipitated with anti-Ikaros were subsequently washed with increasing salt concentrations, which revealed that the interaction of Ikaros with Ezh2 and Mta2 remained stable at up to 1 M NaCl (Fig. 7d), although the Ikaros–Mta2 interaction appeared to be more resistant. Thus, Ikaros forms stable complexes with PRC2. To determine whether specific Ikaros domains were required for interaction with PRC2, we generated ILC87 cells constitutively expressing Ikaros proteins deleted for the amino-terminal domain (ΔN, aa1–114), the DNA-binding domain (ΔDBD, aa119–223) or the dimerization domain (ΔDIM, aa457–508). After IP with anti-Ikaros, the ΔN and ΔDBD, but not the ΔDIM, proteins interacted with Ezh2 (Fig. 7e), suggesting that dimerization may be important for PRC2 interaction.

To determine whether Ikaros interacts with PRC2 in primary thymocytes, nuclear extracts from WT DN thymocytes were immunoprecipitated with antibodies against Ikaros or Suz12 and analysed for Ikaros, Ezh2 and Suz12 (Fig. 7f). Both Ezh2 and Suz12 co-immunoprecipitated with Ikaros, whereas Ikaros and Ezh2 co-immunoprecipitated with Suz12. These results demonstrate that Ikaros interacts with PRC2 in DN thymocytes.

The NuRD complex has been reported to be important for PRC2 recruitment and activity in other systems^30^,^31^,^32^,^33^,^34^. Our data, however, suggest that PRC2 does not interact with NuRD in the present system. We further evaluated whether the Ikaros–PRC2 interaction required NuRD, by first depleting nuclear extracts from ILC87 cells constitutively expressing Ikaros of NuRD-associated proteins, or not, with antibodies against Mta2 and Mi2β (Fig. 7g). Although Mi2β was nearly absent from the depleted extracts, Mta2 was reduced approximately twofold (Supplementary Fig. 9 lane 3). Samples were then immunoprecipitated with antibodies specific for Ikaros, Mta2 or Mi2β and analysed for Ezh2, Suz12, Mta2, Mi2β or Ikaros. As expected, depletion of Mta2 and Mi2β diminished the abundance of these proteins in the precipitated samples (Fig. 7g lanes 4, 6 and 8). However, the Ezh2 and Suz12 levels in the anti-Ikaros precipitated samples were unaffected by the Mta2/Mi2β depletion (Fig. 7g lanes 3 and 4), indicating that the Ikaros–PRC2 interaction was still intact. In addition, Ezh2 and Suz12 were not detected in the anti-Mta2 or anti-Mi2β precipitated samples (Fig. 7g lanes 5–8), suggesting a lack of interaction between PRC2 and NuRD proteins. These results indicate that Ikaros forms distinct complexes with PRC2 and NuRD.

### Ikaros and PRC2 co-localize at genomic sites lacking NuRD

**Figure 8:**
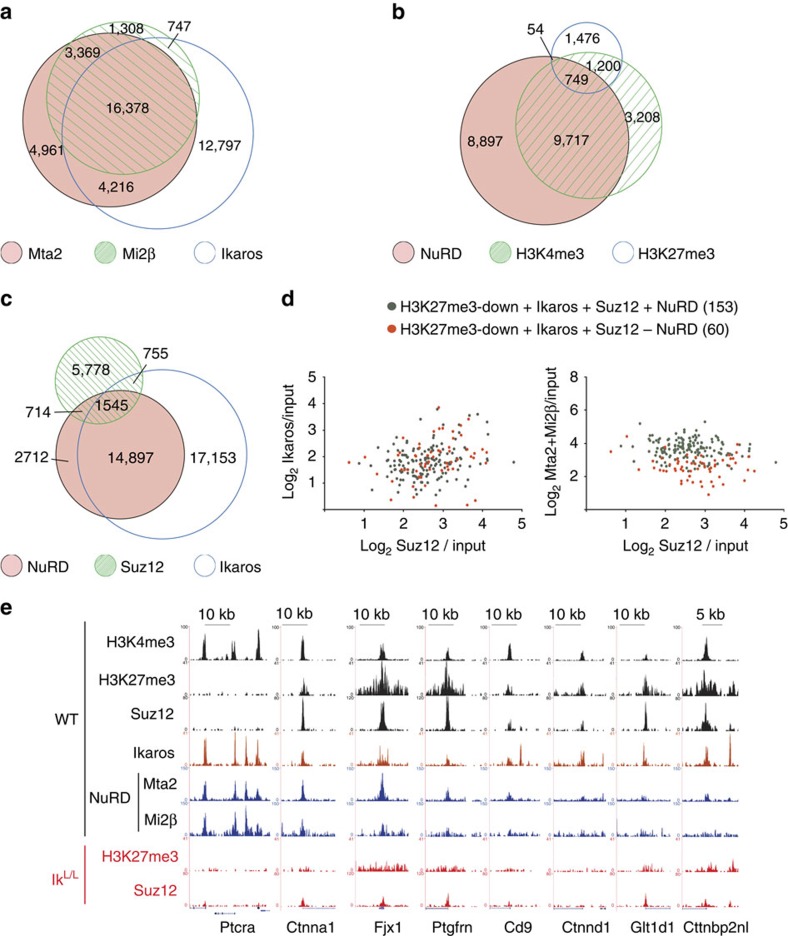
Ikaros and PRC2 co-localize at genomic regions devoid of NuRD. ChIP-seq analysis of Mta2, Mi2β, H3K4me3 and H3K27me3 (Fig. 2), Ikaros (Fig. 3) and Suz12 (Fig. 4) in WT DN3 thymocytes. Venn diagrams of (**a**) 43,776 merged genomic regions bound by Mta2, Mi2β and Ikaros; (**b**) 25,301 merged genomic regions occupied by NuRD (that is, Mta2 and Mi2β), H3K4me3 and H3K27me3; (**c**) 43,554 merged genomic regions bound by NuRD, Ikaros and Suz12. It is noteworthy that each merged genomic region may comprise several overlapping binding sites from the different samples. (**d**) Scatter plot comparisons of Ikaros (left) and NuRD (right) binding to 213 Suz12- and Ikaros-bound regions that exhibit decreased H3K27me3 in Ik^L/L^ thymocytes (Supplementary Fig. S6b,c; it is noteworthy that 3 of the 213 common Ikaros/Suz12-bound regions considered here had 2 distinct Suz12 peaks, of which only the ones with higher tag numbers were kept for this analysis). Binding intensities are expressed as log_2_ FC over input. Regions bound or not by NuRD are shown in grey and red, respectively. (**e**) Representative genome browser tracks illustrating occupancy by Ikaros, Suz12 and NURD, as well as H3K4me3 and H3K27me3 levels of selected loci. H3K27me3 and Suz12 tracks from Ik^L/L^ DN3 thymocytes are shown at the bottom. The *Ptcra* locus is shown as an active gene with high levels of NURD, Ikaros and H3K4me3.

To determine whether Ikaros co-localizes with PRC2 independently of NuRD *in vivo*, we evaluated Mta2 and Mi2β binding, along with H3K4me3 (which co-localizes with NuRD in DP cells)^6^, in DN3 thymocytes and compared these with Ikaros, Suz12 and H3K27me3. Mta2 and Mi2β overlapped at 19,747 sites (hereafter referred to as NuRD sites) and were detected alone at 9,177 regions for Mta2 and 2,055 regions for Mi2β (Fig. 8a). Most of the NuRD-bound regions were also bound by Ikaros (*n*=16,378), in agreement with published data^6^. H3K4me3 frequently co-localized with NuRD (10,466 sites, 70% of H3K4me3 islands), but H3K27me3 and NuRD co-localization was remarkably less frequent (803 sites, 23% of H3K27me3 islands) and occurred almost exclusively on bivalent H3K4me3/H3K27me3 domains (749 sites; Fig. 8b). Similarly, NuRD was observed at only 25% of Suz12 sites (2,259 of 8,792 regions; Fig. 8c). Importantly, Ikaros co-localized with Suz12 on 755 regions that lacked detectable NuRD. These results support our biochemical data that Ikaros interacts with PRC2 independently of NuRD.

Finally, we asked whether NuRD co-localizes with Ikaros and Suz12 at loci that were dependent on Ikaros for H3K27 trimethylation (Fig. 8d,e). Of the 213 loci where overlapping Ikaros/Suz12 binding was detected, 153 were occupied by NuRD (for example, *Ctnna1*, *Fjx1* and *Ptgfrn*), whereas 60 were not (for example, *Cd9*, *Ctnnd1*, *Glt1d1* and *Cttnbp2nl*). In addition, there was no correlation between the presence or absence of NuRD and the intensity of Ikaros or Suz12 binding. Thus, Ikaros can act independently of NuRD to regulate PRC2 activity on a number of target genes, to establish the H3K27me3 mark in DN3 cells.

## Discussion

We show that Ikaros regulates the epigenetic silencing of >500 developmentally regulated loci in CD4^−^CD8^−^ thymocytes via PRC2, which include genes specifically expressed in HSCs or activated by Notch. Many genes affected by Ikaros exhibit bivalent epigenetic marks in WT cells and are derepressed in Ikaros-deficient cells through the selective loss of H3K27me3. Ikaros binding results in PRC2 recruitment and H3K27 trimethylation. Further, Ikaros complexes with PRC2 in T-cell lines and primary thymocytes, independently of NuRD. These results identify a fundamental mechanism of Ikaros as a regulator of PRC2 function in T lymphocytes.

Interestingly, our data suggest that PRC2 activity is not required for the downregulation of Ikaros target gene expression, which occurs in the presence of a PRC2 inhibitor. As most genes with Ikaros-dependent H3K27me3 increases also showed decreased expression, it is possible that transcriptional repression is a prerequisite for PRC2 tethering, as was recently suggested^35^. Ikaros target genes may require PRC2 and H3K27 trimethylation to maintain repression through epigenetic memory, in particular in DP thymocytes where Ikaros can no longer be detected at the DNA. Nonetheless, repression alone does not ensure PRC2 recruitment, as Ikaros represses the expression of other genes that do not gain H3K27me3, suggesting a role for additional mechanisms.

The majority of the genes affected by the loss of H3K27me3 are normally expressed in HSCs and silenced during T-cell differentiation, and the top biological functions associated with them are cell/organ morphology and development (Supplementary Table 3). These data implicate Ikaros as a negative regulator of the HSC gene expression programme in early T cells. Interestingly, HSC-specific genes were previously found to be inefficiently silenced in lymphoid-committed progenitors of Ikaros-null mice^4^, but only 3 (*Ppic*, *Rhoj* and *Socs2*) of the 22 genes identified in that report were deregulated in our study, suggesting that Ikaros regulates distinct target gene repertoires in progenitor cells versus thymocytes. The persistent expression of a stem cell gene repertoire may also be associated with the tumour suppressor function of Ikaros. *IKZF1* mutations are found in 12% of early T-cell precursor ALL, which are also characterized by mutations in PRC2 genes (*SUZ12*, *EZH2* or *EED*) in 42% of cases^36^. Transcriptomic signatures of human B-ALL or myeloid leukemias associated with *IKZF1* mutations have also been shown to be enriched in HSC-related genes^37^,^38^.

Ikaros and PRC2 also repress the expression of some Notch target genes (*Hes1*, *Mpzl2* and *Scn4b*), which are transiently induced in DN2 and DN3 cells, and require Ikaros to be efficiently silenced in DN4 and DP cells^5^. Ikaros binds to these genes in DN3 cells (see Fig. 3b for *Mpzl*2 and *Scn4b*), perhaps setting the stage for later PRC2 recruitment in DN4 and DP cells when Notch signalling is no longer required. Intriguingly, genes (*Lmcd1*, *Fjx1* and *Myo1b*) activated by Notch only in the context of Ikaros deficiency^5^ show striking decreases in H3K27me3, suggesting that loss of this mark defines a permissive epigenetic state in the mutant cells.

Given the various facets of PRC2 regulation by Ikaros, it will be important to investigate whether Ikaros influences PRC2 function during peripheral T-cell development. For example, Ikaros has been reported to positively regulate T-helper 2 (Th2) polarization^8^, in part by repressing *Tbx21* expression^9^, which is required for Th1 development. Interestingly, PRC2 has been shown to bind and prevent the ectopic expression of *Tbx21* in differentiating Th2 cells^39^. As H3K27me3 levels are strongly decreased at the *Tbx21* locus in Ik^L/L^ DN and DP cells (Supplementary Fig. 2b), epigenetic regulation of *Tbx21* by Ikaros via PRC2 might also control its function in Th cell differentiation. In addition, Ikaros may influence PRC2 activity in other cell types, as some of the genes identified here (*Ctnnd1*, *Mmp14*, *Vwf*, *Dock1* and *Hes1*) are strongly upregulated in Ikaros-deficient dendritic cells^40^ and Ikaros is important for PRC2 recruitment to *Hes1* in erythrocytes^41^.

Our data shed light on how PRC2 is targeted to chromatin. This question remains poorly understood and several mechanisms including transcription regulators, non-coding RNAs and non-methylated non-transcribed CpG regions have been implicated^42^. The most compelling evidence for a role of transcription factors was obtained in *Drosophila*, where factors such as pleiohomeotic and sex-comb-on-midlegs target PRC2 to DNA^43^. Nonetheless, several studies have also implicated DNA-binding factors in mammalian cells, notably: YY1 in mesenchymal stem cells^44^, REST in neuronal progenitors^45^,^46^ and Foxp3 in regulatory T cells^47^. We propose that Ikaros mediates PRC2 recruitment in thymocytes.

One common feature of the Ikaros–PRC2 binding sites is the absence of motifs for other regulators, such as E2A, Runx1 and Ets1, which are often found near Ikaros motifs. This lack of recognition sequences was previously suggested to be an important property of the GC-rich sequences responsible for PRC2 binding^48^. Ikaros binding at these regions was also different, as it was spread over long stretches of DNA rather than a short distance typical of other Ikaros peaks. Indeed, these regions were characterized by numerous Ikaros motifs, suggesting that they contain low affinity binding sites. It is worth noting that, even though the number of loci at which Ikaros controls PRC2 recruitment may be low when compared with the total Ikaros sites, it is in the same range as the number of loci affected by Foxp3 or REST^46^,^47^. Ikaros may also act redundantly with other factors or gene-specific features to modulate PRC2 binding. For example, Ikaros and Suz12 both bind to *Lmo2* and *Tal1* in DN3 cells, but Suz12 binding is unaffected by Ikaros deficiency.

Previous studies have implicated the NuRD complex in Polycomb function^30^,^31^,^32^,^33^,^34^. Our results, however, suggest that Ikaros can mediate PRC2 binding independently of NuRD, as we show a strong functional interaction between Ikaros and PRC2, the existence of distinct Ikaros–NuRD and Ikaros–PRC2 complexes, and the absence of NuRD at ∼30% of common Ikaros–PRC2 target sites. Further investigation will be required to clarify the relevance of NuRD at other sites.

Loss of H3K27me3 was previously observed in Ikaros-null DP cells^6^. However, as Ikaros did not bind the affected loci in WT DP thymocytes and as *de novo* Mi-2β binding was observed for many of these sites in mutant cells, it was concluded that the reduction in H3K27me3 was an indirect consequence of NuRD redistribution in the absence of Ikaros. In contrast, our results suggest that Ikaros directly affects PRC2 binding and the appearance of H3K27me3 marks in DN cells, the latter of which is maintained in DP thymocytes even after Ikaros binding is no longer detected. It remains unknown whether other Ikaros family members can contribute to H3K27me3 maintenance, in particular Aiolos, which is induced in DP cells. Interestingly, the published Aiolos ChIP-seq data from Ikaros-null thymocytes^6^ showed Aiolos binding to ∼50% of the affected loci identified in our study, suggesting that Aiolos cannot rescue H3K27 trimethylation in DP cells.

In conclusion, our study reveals a novel mechanism by which Ikaros represses gene expression in T cells. We anticipate that the Ikaros–PRC2 interaction will play a major role in lymphoid differentiation and tumour suppression.

## Methods

### Cell lines

The ILC87 cell line was previously described^5^. To retrovirally express inducible forms of the full-length or truncated Ikaros proteins, the ERT2 complementary DNA encoding the ligand-binding domain of the ER^49^ was fused to the 3′-ends of the cDNAs encoding the Ikaros1 isoform (Ik1)^5^ or Ik1 proteins lacking residues 1–114 (ΔN), 119–223 (ΔDBD) or 457–508 (ΔDIM) and cloned into the MigR1 vector upstream of the internal ribosome entry site that precedes a GFP coding sequence. ILC87 cells were transduced and GFP^+^ cells were sorted to generate stable cell lines. 4OHT (Sigma) was used at a final concentration of 100 nM and the Ezh2 inhibitor EPZ005687 (Selleckchem) at 2.5 μM. To generate ILC87 cells constitutively expressing Ik1, Ik1 cDNA was cloned into the MigR1 vector and ILC87 cells were first transduced with pMCSV-mBcl2-DsRed^50^ to increase cell survival, and then with the MigR1 constructs, and DsRed^+^ GFP^+^ cells were sorted and stable cultures were established.

### Mice

Ik^L/L^ and Ik^f/f^ CD4-Cre tg mice were described previously^5^,^16^. Ik^f/f^ mice^51^ were also crossed with Lck-Cre tg mice^52^. Ik^L/L^ mice were on the C57Bl/6 background, whereas Ik^f/f^ CD4-Cre^+^ and Ik^f/f^ Lck-Cre^+^ animals were on a mixed C57Bl/6-129Sv background. All mice used here were 3–4 weeks old and were both male or female. All animal experiments were approved by the IGBMC ethical committee (Com'Eth #2012-092).

### Cell purification

Thymocytes were depleted of CD4^+^ and CD8^+^ cells using sheep anti-rat IgG-conjugated Dynabeads (Invitrogen). The remaining cells were stained for lineage (Lin) markers (CD4, CD8, CD3, B220, CD11b, Gr1 and NK1.1), CD44, CD25 and 4,6-diamidino-2-phenylindole (DAPI). DAPI^−^ DN1 (Lin^−^CD25^−^CD44^+^), DN2 (Lin^−^CD25^+^CD44^+^), DN3 (Lin^−^CD25^+^CD44^−^) and DN4 (Lin^−^CD25^−^CD44^−^) cells were sorted. To isolate DP cells, DAPI^−^CD4^+^CD8^+^CD3^low^ cells were sorted.

For LSK cells, bone marrow cells were depleted for Lin^+^ (B220, CD11b, Gr1 and Ter119) cells with anti-rat IgG-conjugated Dynabeads. The remaining cells were first stained for CD16/CD32, Lin, Sca1, c-Kit and DAPI. DAPI^−^ LSK cells were sorted. Cell sorting was performed on a FACSAria II SORP (BD Biosciences). Purity was >98%.

DP thymocytes were also isolated by positive selection of CD8^+^ cells (Mitenyi Biotech). Ninety-two to 95% of the isolated cells were CD4^+^CD8^+^. Similar results were obtained using DP populations purified by cell sorting or by magnetic bead selection. Total DN thymocytes were depleted for CD4-, CD8- and Ter119-positive cells, and then further depleted for CD4-, CD8-, CD3-, B220-, CD11b-, Gr1-, NK1.1- and Ter119-positive cells with anti-rat IgG-conjugated Dynabeads. Isolated cells contained 70–80% cells with a DN1–4 profile. Antibodies were from BD Biosciences and eBioscience.

### Antibodies

The purified rabbit polyclonal carboxy terminus-specific anti-Ikaros antibody and the mouse monoclonal anti-ER and anti-Cre antibodies were generated in-house. The N terminus-specific anti-Ikaros (sc-13039, Santa Cruz), anti-Suz12 (3737, Cell Signaling; sc46264, Santa Cruz), anti-Ezh2 (3147S, Cell Signaling), anti-Mta2 (ab8106, Abcam), anti-Mi2β (CHD4; ab70469, Abcam), anti-H3K27me3 (07-449, Millipore), anti-H3K4me3 (ab8580, Abcam), anti-H3 (06-755, Millipore; ab1791 Abcam), anti-H4 (ab7311, Abcam) and anti-β-actin (A5441; Sigma) antibodies were purchased.

### Chromatin immunoprecipitation

The ChIP protocol was adapted from the Millipore ChIP Assay Kit (17-295) with minor modifications. Cells were washed in PBS and 2–6 × 10^7^ cells were cross-linked at 37 °C for 10 min in 5 ml PBS/0.5% BSA/1% ultra-pure formaldehyde (Electron Microscopy Sciences). Quenching with 125 mM glycine and a cold PBS wash (containig 1 × protease inhibitor cocktail (PIC); Roche) was followed by cell lysis in 5 ml of 1% Triton X-100, 50 mM MgCl_2_, 100 mM Tris-HCl pH 7.1, 11% sucrose, 1 × PIC for 10 min on ice. Nuclei were pelleted and were lysed in 500 μl of 1% SDS, 50 mM Tris-HCl, 10 mM EDTA, 1 × PIC. Chromatin was sonicated to 500–300 bp using a Bioruptor 200 (Diagenode), cleared by centrifugation and sonication efficiency was verified. Sonicated chromatin diluted 4 × with 0.01% SDS, 1.1% Triton X-100, 1.2 mM EDTA, 16.7 mM Tris-HCl pH 8.1, 167 mM NaCl_2_, 1 × PIC was pre-cleared with 100 μl protein A sepharose 50% slurry or with 50 μl Magna ChIP Protein A Magnetic Beads (Millipore) previously blocked with 0.5% BSA. Cell equivalents (2.5 × 10^5^–30 × 10^6^) were diluted 2.5 × in the same buffer and incubated overnight (ON) with 5 μg (for anti-Ikaros (home-made), anti-Mta2 (ab8106), anti-Mi2β (ab70469), anti-H3K27me3 (07-449), anti-H3K4me3 (ab8580), anti-H3 (06-755)) or 5 μl (for anti-Suz12 (3737)) test antibodies, or 5 μg IgG control, except for Fig. 3d, where 1.5 × 10^5^ cell equivalents were incubated with 2 μg anti-H3K27me3 (07-449), anti-H3 (06-755)) or IgG. Protein–DNA complexes were bound to 75 μl 50% protein A slurry or 30 μl Protein A Magnetic Beads for 5–6 h at 4 °C and washed 1 × with low-salt buffer (20 mM Tris-HCl pH 8.1, 150 mM NaCl_2_, 2 mM EDTA, 1% Triton X-100, 0.1% SDS), high-salt buffer (20 mM Tris-HCl pH 8.1, 500 mM NaCl_2_, 2 mM EDTA, 1% Triton X-100, 0.1% SDS), LiCl buffer (10 mM Tris-HCl pH 8.1, 1 mM EDTA, 1% deoxycholate, 1% NP40, 0.25 M LiCl) and Tris EDTA (10 mM Tris-HCl pH 8, 1 mM EDTA). Samples were eluted, cross-linking was reversed and DNA was purified using the iPure Kit (Diagenode).

### ChIP sequencing

Libraries were prepared according to standard Illumina protocols and were validated with the Agilent Bioanalyzer. Single, 36-bp read sequencing runs were performed on an Illumina GAIIx, except for the Suz12, Mta2 and Mi2β ChIP-seq for which single, 50-bp reads were sequenced on a HiSeq2000. Image analysis and base calling was performed with the Illumina pipeline and reads were aligned to the mm9 mouse genome with Bowtie^53^. ChIP-seq tag libraries from two experiments were combined for the analysis of WT DN3, DN4 and DP H3K27me3, and input samples. For visualization in the UCSC genome browser, either Wig files were generated by extending the reads to 200 bp length and the read densities in 25 bp bins were normalized to the library size, or quantile-normalized 200 bp binned BedGraph tracks were used. All analyses were performed with unique reads that did not overlap with >5 bp with known short or long interspersed nuclear elements, long terminal repeats and satellites. Wig tracks of the Suz12 data in ILC87-Ik1-ER cells (Fig. 5a) were also normalized by the 10% trimmed mean peak-tag counts.

*Peak callings and filtering*. All peak calling analyses were performed with unique reads that did not overlap with >5 bp with known short or long interspersed nuclear elements, long terminal repeats and satellites. H3K27me3 and H3K4me3 peak callings were performed using SICER v1.1 (ref. 54) with the following settings for the analysis of DP cells (Fig. 1 and Supplementary Fig. 1): window size=200; fragment length=200, *E*-value=100, window *P*-value=0.02, false discovery rate=10^−3^ and gap size set to 1,400 for H3K27me3 and 800 for H3K4me3, respectively. For the analysis of H3K27me3 on thymocyte subsets, LSK cells (Fig. 2 and Supplementary Fig. 2) and ILC87-Ik1-ER cells (Fig. 5), window size=200; fragment length=300, *E*-value=0.1, window *P*-value=0.004, false discovery rate=10^−3^ and gap size=1,200 were used. The same settings, except for gap size=600, were used for the H3K4me3 data from DN3 cells (Fig. 8). For the data from DP cells (Fig. 1 and Supplementary Fig. 1), eligible islands were required to be min 800 bp for H3K27me3 and 400 bp for H3K4me3, to have >50 tags and a tag number/island length value >0.03 for H3K27me3 and >0.04 for H3K4me3 in the merged genomic regions. Only the most TSS-proximal islands were considered per gene. For primary thymocyte subsets and LSK cells (Figs 2 and 8, and Supplementary Fig. 2; H3K27me3 and H3K4me3), islands identified by SICER were further filtered to be ≥800 bp long, with SICER island scores >45 and the tag/length ratio >0.04. H3K27me3 islands corresponding to merged regions with the following characteristics were excluded from further analysis: regions ≤1.2 kb that are >5 kb away from the closest TSS (to eliminate small intergenic ‘islands' that probably result from sequencing noise), regions <2 kb that are in close vicinity (<5 kb) to a longer (≥3 × ) island (to prevent the selection of island ‘tails' as separate islands) and merged regions in which the input's normalized tag/length ratio is >0.024. MACS v1.4 (ref. 55) was used for peak calling on Ikaros, Suz12, Mta2 and Mi2β ChIP-seq samples using default parameters with the following modifications: band width was provided as the fragment length of the libraries according to the Agilent Bioanalyzer and *P*-value cutoff was set to 10^−7^.

*Annotation, quantification and normalization*. Merged genomic regions of overlapping peaks across samples were created using mergeBed from the BEDTools suite (2.16.2)^56^. The annotations and tag numbers were determined by the HOMER software^57^. Promoters were defined as regions between −10 and 1 kb relative to TSS; other annotated features (untranslated regions, introns, exons, transcription termination sites and non-coding) downstream of the 1 kb position were commonly referred to as gene body (intragenic) regions; and all other genomic positions were labelled intergenic. Tag numbers for all data sets were normalized to the library sizes. For fold-change comparisons, pairwise normalization between WT and mutant samples were done as follows: for DP samples (Fig. 1 and Supplementary Fig. 1), the 10% trimmed means were used to normalize the larger data set; for the data in Fig. 2 and Supplementary Fig. 2, linear regression lines were calculated for each WT/mutant pair, and the equation of the line was used for normalization. HOMER was used to generate coverage plots and the data matrices for tag density heatmaps that were normalized using the 10% trimmed-mean for Figs 1b, 3a and 4a, and Supplementary Fig. 1a. Clustering was performed using Cluster 3.0 and visualized with Java Tree View. For motif analysis of Ikaros peaks, enriched motifs were identified with the MEME software and matched to transcription factor-binding sites in the Jaspar data base with the Tomtom algorithm. The labelling of GGAA-containing motifs as Ikaros motifs derives from ChIP-seq studies for Ikaros in T and B cells^5^,^6^,^24^,^25^,^26^. The identity of the Ets1 motif was inferred from the study by Hollenhorst *et al*.^58^.

### IP of nuclear extracts and western blot analysis

Cells were incubated in hypotonic buffer (10 mM HEPES pH 7.3, 1.5 mM MgCl_2_, 10 mM KCl, 0.5 mM dithiothreitol (DTT), 1 × PIC, 1 × phosphatase inhibitor cocktail (Sigma), 1 mM NaF, 0.5 mM phenylmethyl sulphonyl fluoride) on ice and pelleted. Nuclei were lysed in RIPA buffer (50 mM Tris pH 8.1, 150 mM NaCl_2_, 2 mM EDTA, 1% NP40, 0.5% Na-deoxycholate, 0.1% SDS, 0.5 mM DTT, 1 × PIC, 1 × phosphatase inhibitor cocktail, 1 mM NaF, 0.5 mM phenylmethyl sulphonyl fluoride) for 30 min at 4 °C. Nuclear extracts were cleared by centrifugation. For each IP reaction, 500 μl extract was pre-cleared with 50 μl Protein A Sepharose 50% bead slurry that was previously washed and blocked with 0.5 mg ml^−1^ BSA. Pre-cleared extracts were incubated with 2.5–5 μg anti-Ikaros or 5 μl anti-Suz12 antibodies, or 5 μg rabbit IgG ON at 4 °C. Antibody–protein complexes were captured with 100 μl 50% bead slurry for 5–6 h and washed 5 × at 4 °C with RIPA buffer containing 300 mM NaCl_2_, and subsequently denatured by boiling with SDS–PAGE loading buffer.

To deplete NuRD, a 2.4-ml BSA-blocked half-and-half mix of Protein A and Protein G Sepharose 50% bead slurry was coated with 60 μg anti-Mta2 and 40 μg anti-Mi2β ON at 4 °C and washed 5 × with RIPA buffer (without DTT). Another 2.4 ml Protein A+G mix was coated with 100 μg rabbit IgG as negative control. Two millilitres of nuclear extracts were incubated in three consecutive steps with 0.8 ml of antibody- or 0.8 ml of control IgG-coupled beads for 3 h 2 × and ON 1 × at 4 °C. After incubating with 200 μl BSA-blocked Protein A+G 50% bead slurry for an additional 1 h at 4 °C, supernatants from each were recovered, sampled for input and split into four. IPs on ‘depleted' and control nuclear extracts were performed for 8 h at 4 °C using 100 μl BSA blocked Protein A bead slurry coated with either 5 μg anti-Ikaros, anti-Mta2 or rabbit IgG, or 100 μl Protein G slurry coated with 5 μg anti-Mi2β. Beads were then washed 5 × at 4 °C with RIPA containing 300 mM NaCl_2_ and subsequently denatured by boiling with SDS–PAGE loading buffer.

Proteins were separated on SDS polyacrylamide gels, transferred to polyvinylidene difluoride membranes (Millipore) and detected using horseradish-peroxidase-conjugated secondary antibodies and chemiluminescence (Pierce, Millipore). Primary antibodies were used at 1:1,000 dilution. Western blot images were cropped for presentation. Full-size images are presented in Supplementary Figs 10 and 11.

### Transcriptome analysis

LSK cells were sorted from 6- to 7-week-old WT and Ik^L/L^ mice. RNA was extracted from 5 × 10^4^ cells and used for transcriptome analysis with Affymetrix 430 2.0 arrays using standard amplification methods. LSK transcriptome data were normalized with those from DN3, DN4 and DP cells (GSE 46090) with the Robust Multiarray Average algorithm. K-means clustering was performed using Cluster 3. Transcriptome analysis of the ILC87-Ik1-ER cells treated with ethanol or 4OHT for 24 h was performed with the Affymetrix Gene ST 1.0 arrays.

### GST pull-down assay

The full-length Ik1 isoform was cloned into the pGEXT2 plasmid downstream of the GST tag. *E. coli* BL21 was transformed with pGEX2T-Ik1 or the empty vector expressing GST alone. ON cultures were initiated from single colonies, diluted 50 ×, expanded for ∼2 hours until OD_600_=0.5–0.6 and induced with 1 μM isopropyl-β-D-thiogalactoside at 16 °C for 13 h. Bacteria were pelleted at 5,000*g* for 5 min and lysed in 400 μl per 5 ml of the original culture volume of lysis buffer (50 mM NaH_2_PO_4_ pH 8.0, 300 mM NaCl containing 1 × PIC, 1 mM DTT, 1 mg ml^−1^ lysozyme) for 30 min on ice and sonicated. Lysates were cleared by centrifugation at 14,000 r.p.m. for 20 min at 4 °C and the SN was saved as the soluble protein extract. Glutathione agarose beads (Thermo Scientific) were washed 5 × with 50 mM NaH_2_PO_4_ pH 8.0, 300 mM NaCl and 50 μl of the 50% bead slurry was incubated with 400 μl protein extract for 3 h at 4 °C and washed again 5 ×. The recovery of the GST-Ik1 and GST proteins in the soluble protein extracts and their efficient capture on the beads was validated by SDS–PAGE and Coomassie Blue staining. Fifty microlitres of the 50% bead slurry with immobilized GST-Ik1 or GST, or beads alone, were then incubated with nuclear extracts of Ikaros-null ILC87 cells ON at 4 °C. Beads were washed 5 × with RIPA buffer containing 300 mM NaCl and analysed by western blotting.

### PCR primers

Primers used for ChIP–qPCR and reverse transcriptase-qPCR are listed in Supplementary Table 4.

## Additional information

**Accession numbers:** Microarray and ChIP-seq data generated in this study are available from the GEO database under the accession number GSE61149.

**How to cite this article:** Oravecz, A. *et al*. Ikaros mediates gene silencing in T cells through Polycomb repressive complex 2. *Nat. Commun.* 6:8823 doi: 10.1038/ncomms9823 (2015).

## Supplementary Material

Supplementary Information Supplementary Figures 1-11 and Supplementary Tables 1-4
