# Supplementary material for: Ikaros mediates gene silencing in T cells through Polycomb repressive complex 2
Source: Nat Commun. 2015 Nov 9;6:8823. doi: 10.1038/ncomms9823 (PMC4667618; doi:10.1038/ncomms9823)
Supplement: Supplementary Information — Supplementary Figures 1-11 and Supplementary Tables 1-4 [file ncomms9823-s1.pdf]

Supplementary Figure 1 - Analysis of H3K27me3 and H3K4me3 profiles in WT and Ik$^{L/L}$ DP cells.

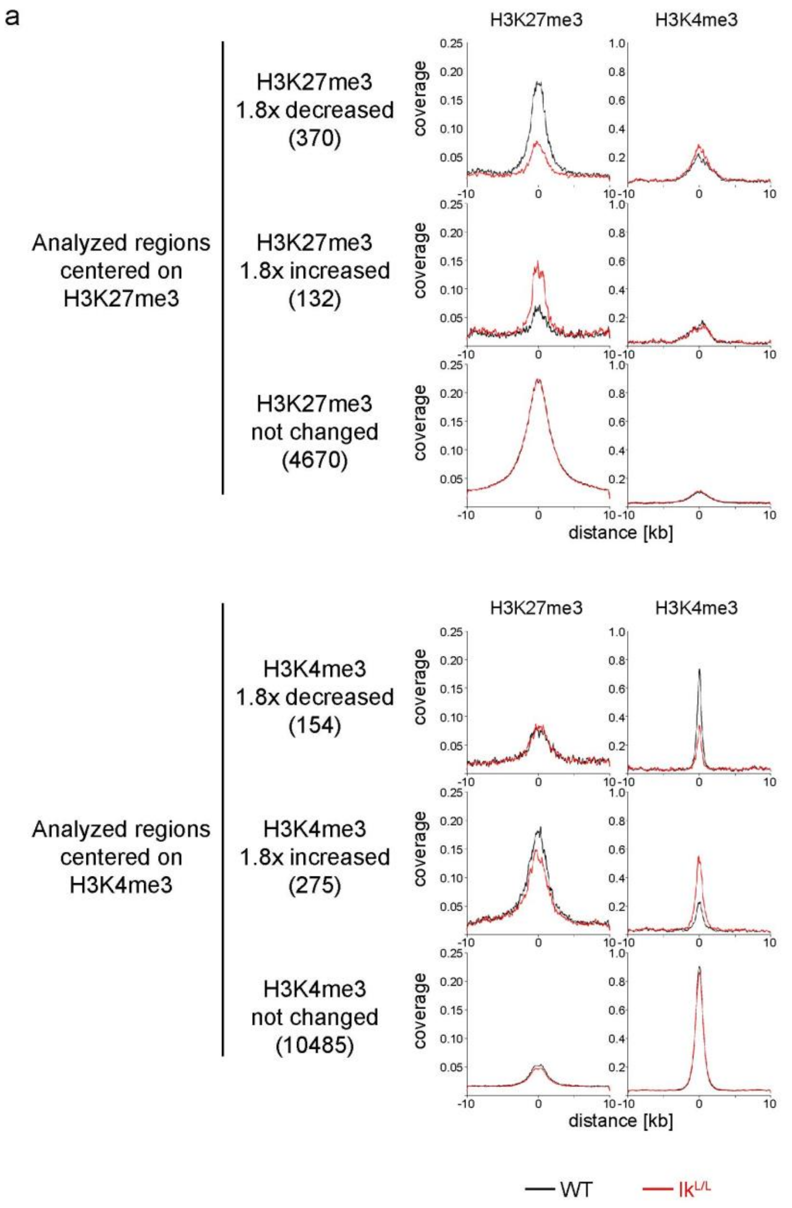

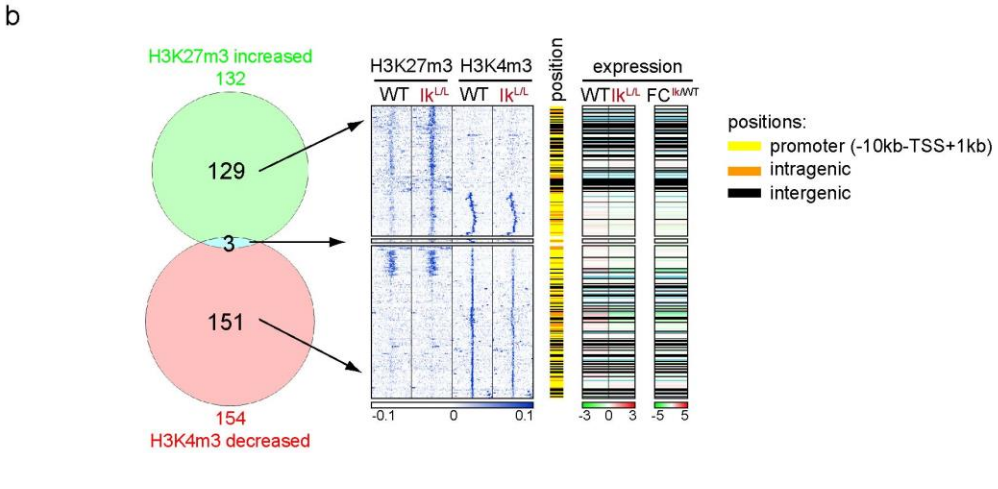

(a) Coverage plots for H3K27me3 and H3K4me3 centered on peaks that were increased/decreased (≥1.8x) or unchanged (<1.8x) in $Ik^{L/L}$ cells, as indicated. Values in parentheses indicate the numbers of islands in each group. Y-axes show the average normalized tag numbers in 20 bp bins 10 kb around the peak centers. **(b)** Venn diagram show the overlap between genomic regions with increased H3K27me3 or decreased H3K4me3 in $Ik^{L/L}$ DP thymocytes. The blue-colored heatmap shows k-means clustering of the tag densities of the corresponding genomic regions for each group. z

Supplementary Figure 2 - Global changes of H3K27me3 in WT and $Ik^{L/L}$ thymocytes during differentiation.

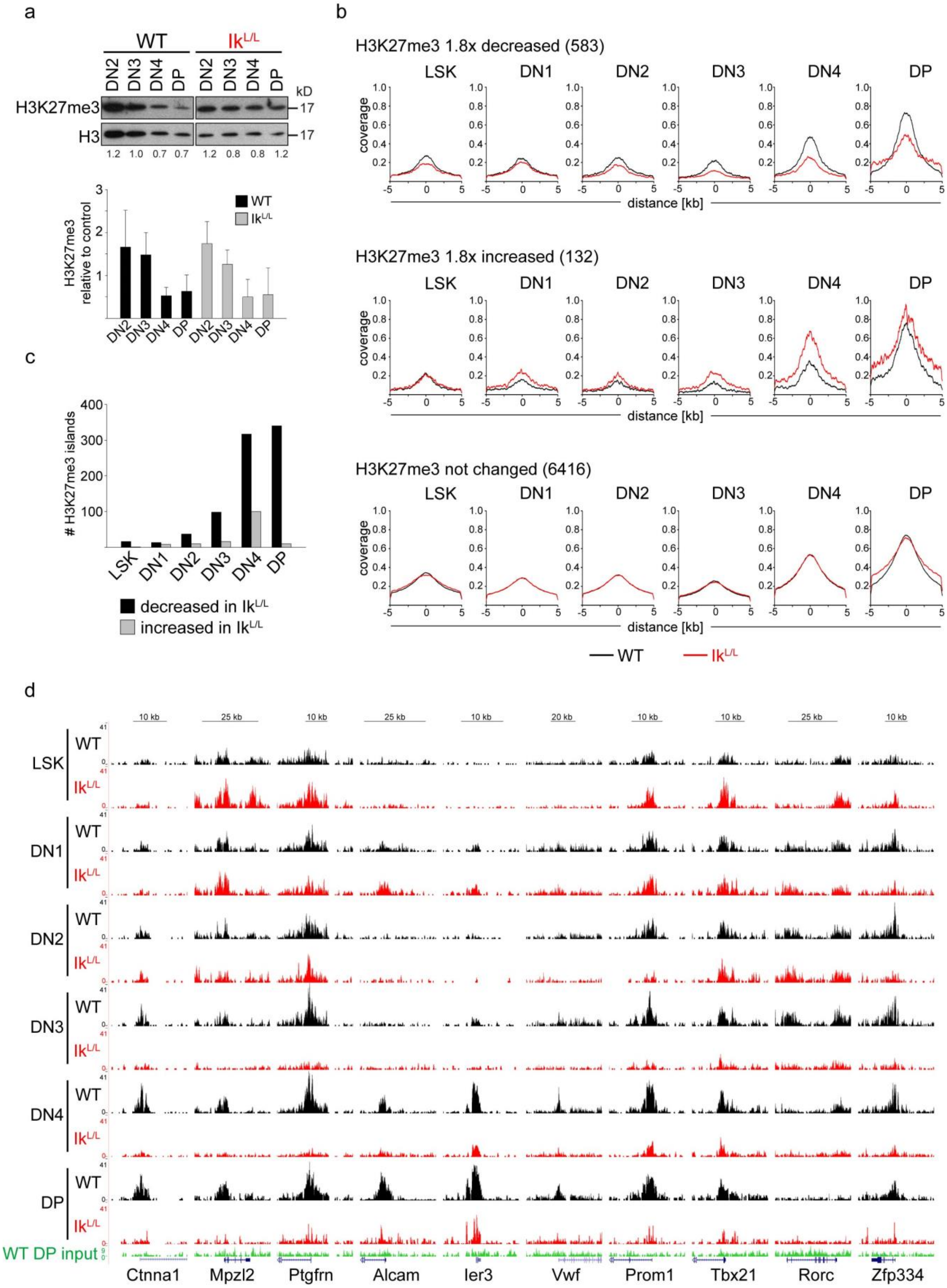

(a) Top**:** Western blot of H3K27me3 in total cell lysates of DN2 to DP thymocytes from WT and $Ik^{L/L}$ mice. Histone H3 is shown as loading control. Numbers indicate the H3K27me3 signals relative to H3, as quantified by ImageJ (v1.44). Bottom: Relative H3K27me3 levels in DN2 to DP thymocytes from WT and $Ik^{L/L}$ mice based on the quantification of n=5 Western blots. Error bars, SD **(b)** Coverage plots of H3K27me3 centered on peaks that are increased/decreased ≥1.8x or unchanged (<1.8x) in the indicated populations. LSK data represent the average of 2 experiments. Values in parentheses indicate the numbers of islands in each group. Y-axes show average normalized tag numbers in 20 bp bins 10 kb around the peak centers. **(c)** Number of H3K27me3 islands that were changed >1.8x in $Ik^{L/L}$ LSK and DN1 to DP cells compared with WT. LSK corresponds to the mean of 2 independent experiments. DP data correspond to islands that were changed in both the sorted DP ChIP-seq experiments (Fig. 2, a and b) and the DP/CD8 experiment (Fig. 1, a-c). **(d)** Representative genome browser tracks of H3K27me3 ChIP-seq data from the indicated cell populations. WT DP input controls in green.

Supplementary Figure 3 - Expression of genes with decreased H3K27me3.

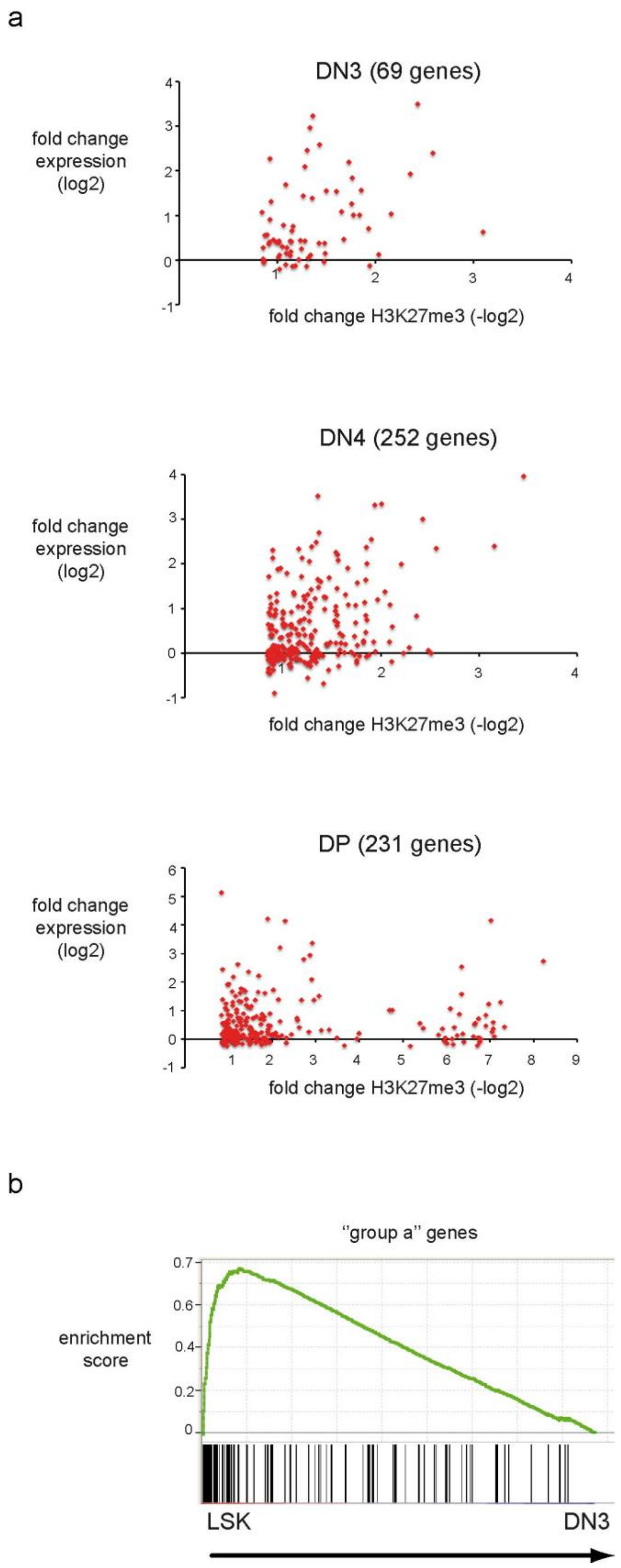

(a) Scatter plots comparing the changes in H3K27me3 levels (x-axis) and the expression of the corresponding genes in the DN3, DN4 and DP subsets (y-axis). Genes that exhibited decreased H3K27me3 levels >1.8x in DN3, DN4 or DP cells were selected, and the corresponding probe sets on the Affymetrix 430 2.0 array identified. Where there were multiple probe sets for a given gene, only the one with the highest absolute fold change was retained. **(b)** Gene set enrichment analysis for the group *a* genes from Fig. 2c, showing their higher expression in LSK cells vs. DN3 cells. Probe sets from the Affymetrix 430 2.0 array were ranked according to the fold change between LSK and DN3 cells.

Supplementary Figure 4 - Ikaros DNA binding profiles during T cell differentiation.

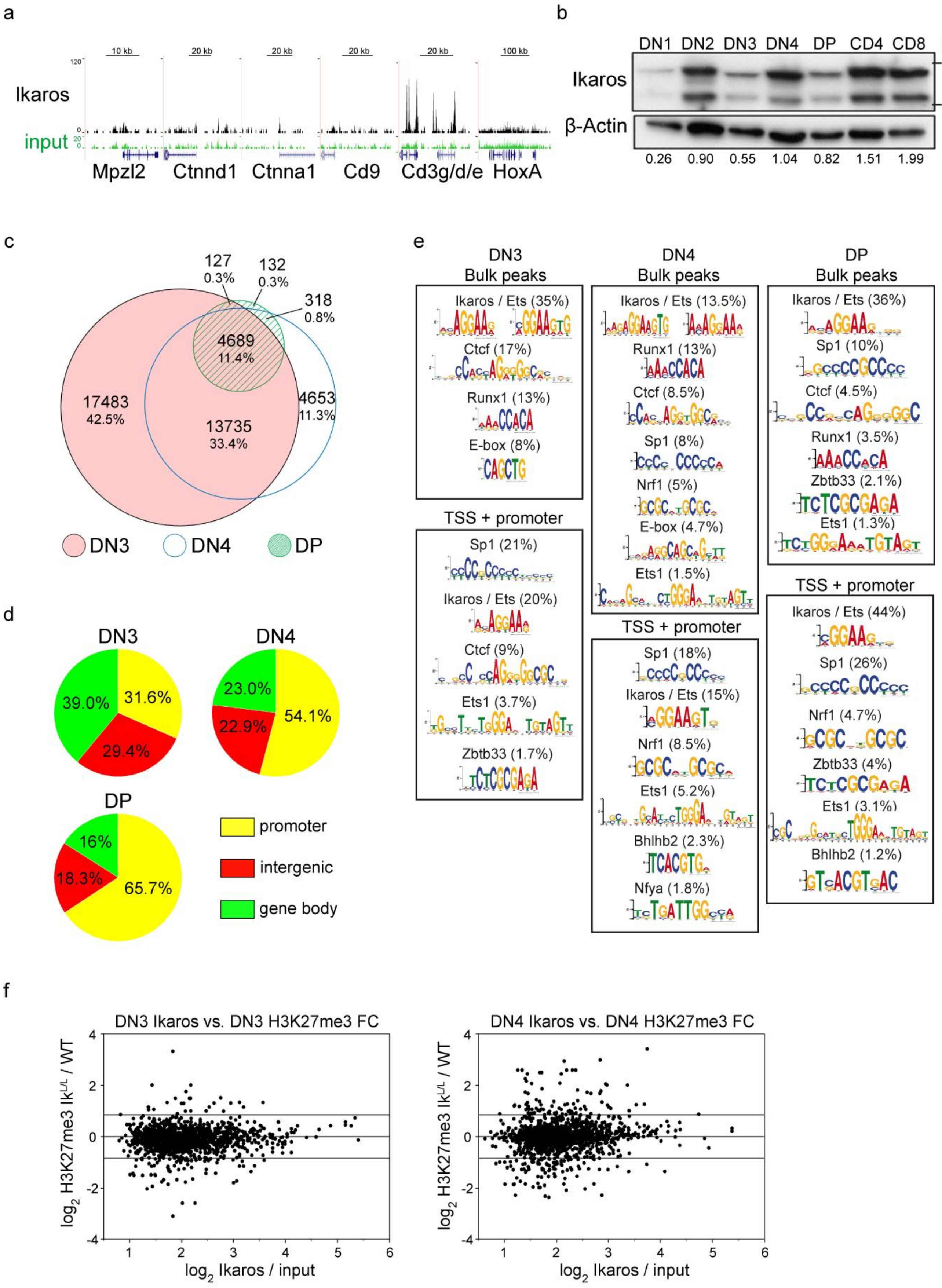

(a) Genome browser tracks of Ikaros ChIP-seq in WT DP cells. Input controls in green. *Cd3d* is shown as a positive control. **(b)** Ikaros protein expression in early thymocytes. Western blots of whole cell lysates of WT 3.5x10^4 (DN1) or 7x10^4 (DN2-4, DP, CD4, CD8) thymocytes. Numbers indicate the Ikaros signals relative to β-actin, as quantified by ImageJ (v1.44). Representative of ≥3 independent experiments. **(c)** Venn diagram of the 41,137 merged genomic regions bound by Ikaros in DN3, DN4 and DP cells (corresponding to either a single peak or multiple peaks per region, as each merged genomic region may comprise multiple overlapping binding sites from the different populations; thus these numbers may not match those in the corresponding text). **(d)** Pie charts depicting the distribution of Ikaros peaks from DN3 (37,050), DN4 (23,656) and DP (5,351) cells among promoter regions (from -10 kb to +1 kb of the TSS), gene bodies or intergenic regions. **(e)** Motif analysis of Ikaros peaks. **(f)** Scatter plots depicting Ikaros enrichment over input (x-axis, $\log_2$) vs $Ik^{L/L}$/WT H3K27me3 FC (y-axis, $\log_2$) in the DN3 and DN4 populations at regions with overlapping Ikaros binding and H3K27me3. The horizontal lines show the threshold used for the selection of differential H3K27me3 levels [$\log_2(1.8)=0.84$].

Supplementary Figure 5 - Characterization of Ik$^{f/f}$ Lck-Cre^{+} mice.

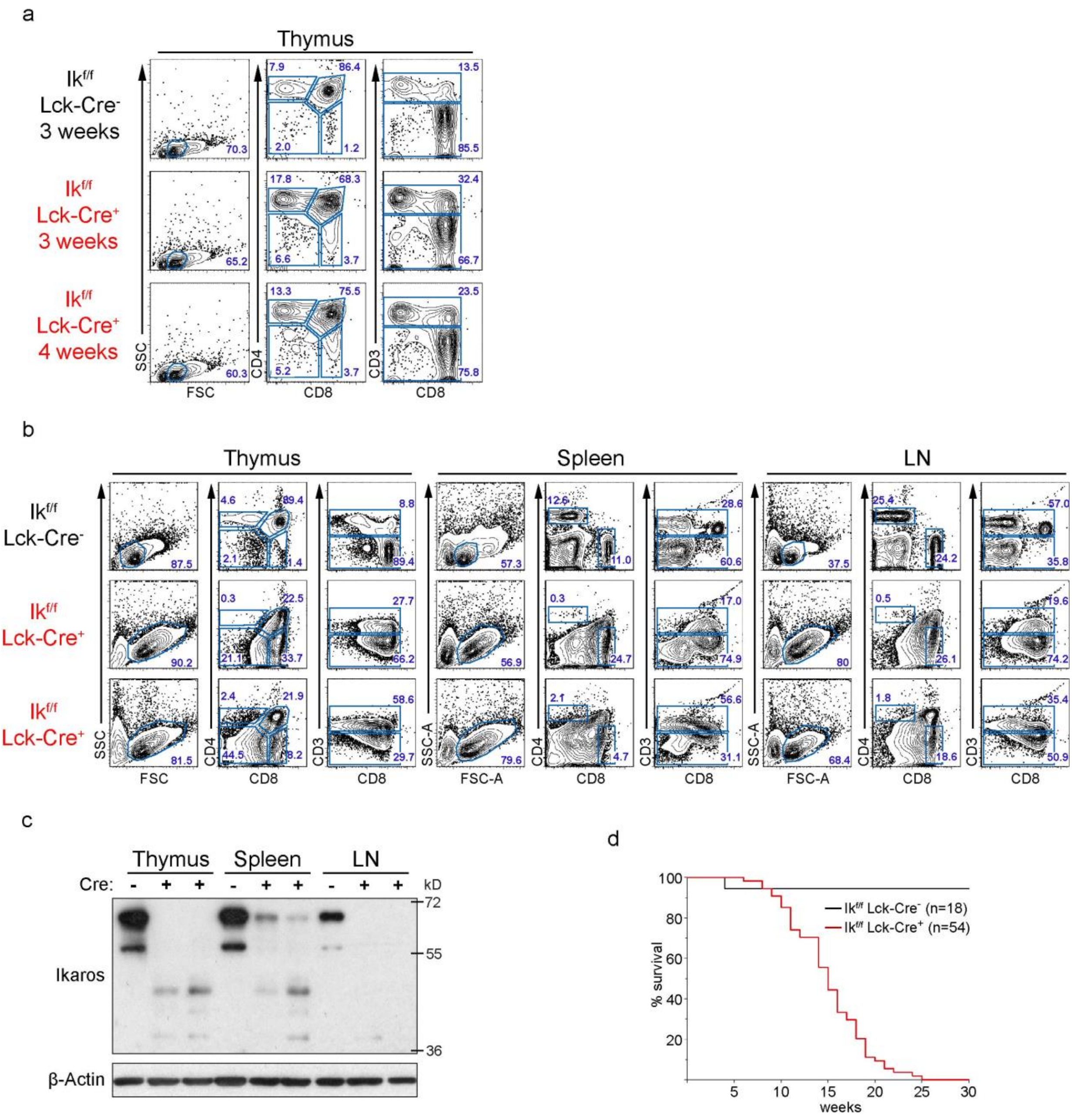

(a) Flow cytometry analysis of thymocytes from 3-wee-old Ik$^{f/f}$ Lck-Cre^{-} and Ik$^{f/f}$ Lck-Cre^{+} mice. Numbers indicate percentage of the gated populations. Mutant mice display an increase in the proportion of mature CD4^{+}CD3^{+} and CD8^{+}CD3^{+} cells. **(b)** Analysis of the thymus, spleen and lymph nodes (LN) of moribund Ik$^{f/f}$ Lck-Cre^{+} mice with T cell leukemia and a healthy Ik$^{f/f}$ Lck-Cre^{-} mouse of similar age (~4 months). Numbers indicate percentage. **(c)** Western blots of total cell lysates from the indicated organs from the mice illustrated in (b), analyzed with an anti-Ikaros antibody specific for the non-targeted N-terminal part of Ikaros. **(d)** Survival curves of Ik$^{f/f}$ Lck-Cre^{-} and Ik$^{f/f}$ Lck-Cre^{+} mice.

Supplementary Figure 6 - Decreased Suz12 binding in $Ik^{L/L}$ DN3 thymocytes at Ikaros target genes.

a

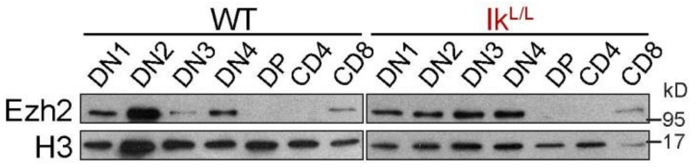

b

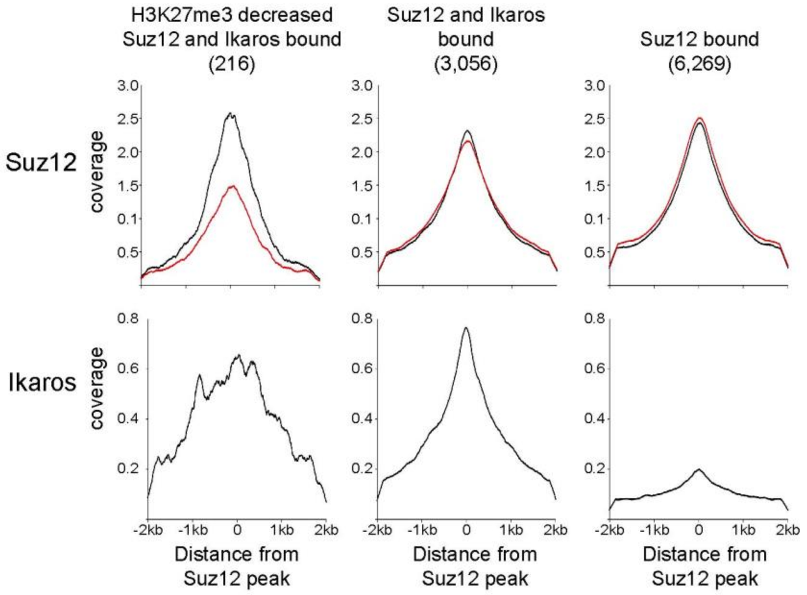

c

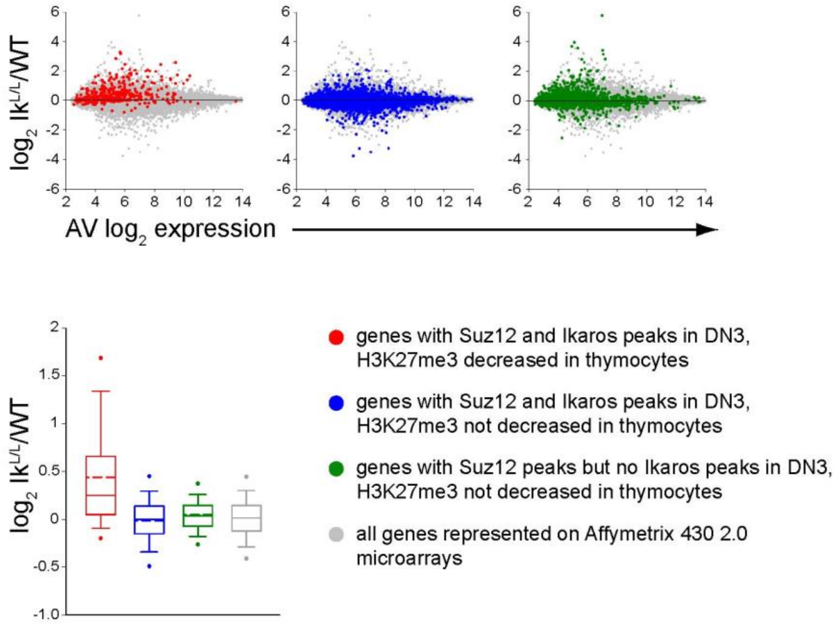

(a) Western blots of whole cell lysates for Ezh2 abundance in WT and $Ik^{L/L}$ thymocyte subsets. Representative of 3 independent experiments. **(b)** Coverage plots of Suz12 peaks from WT (black) and $Ik^{L/L}$ (red) DN3 cells, and Ikaros peaks from WT DN3 cells, centered on 9,541 Suz12 peaks (±2 kb) which were divided into: (left) 216 Suz12 peaks overlapping with Ikaros ($p \leq 10^{-7}$) and H3K27me3 in WT cells at positions where H3K27me3 was decreased in $Ik^{L/L}$ thymocytes; (middle) 3,056 Suz12 peaks overlapping with Ikaros where H3K27me3 was not decreased in $Ik^{L/L}$ thymocytes; (right) the rest of the Suz12 peaks (6,269) that did not overlap with Ikaros. **(c)** Scatter plots and box plot analysis of Affymetrix gene expression data corresponding to the 3 groups of Suz12 peaks in (b). Red corresponds to the 216 Suz12 peaks in (b) which mapped to 187 genes that were represented by 430 probe sets. Blue corresponds to the 3,056 Suz12 peaks in (b) which mapped to 2,500 genes that were represented by 4,868 probe sets. Green corresponds to the 6,269 Suz12 peaks in (b) which mapped to 3,380 genes that were represented by 5,423 probe sets. Gray shows all of the probe sets present on the Affymetrix 430 2.0 microarrays. In the box plots, dashed lines indicate means, dots indicate the 5th and 95th percentiles.

Supplementary Figure 7 - Ik1-ER expression in ILC87 cells.

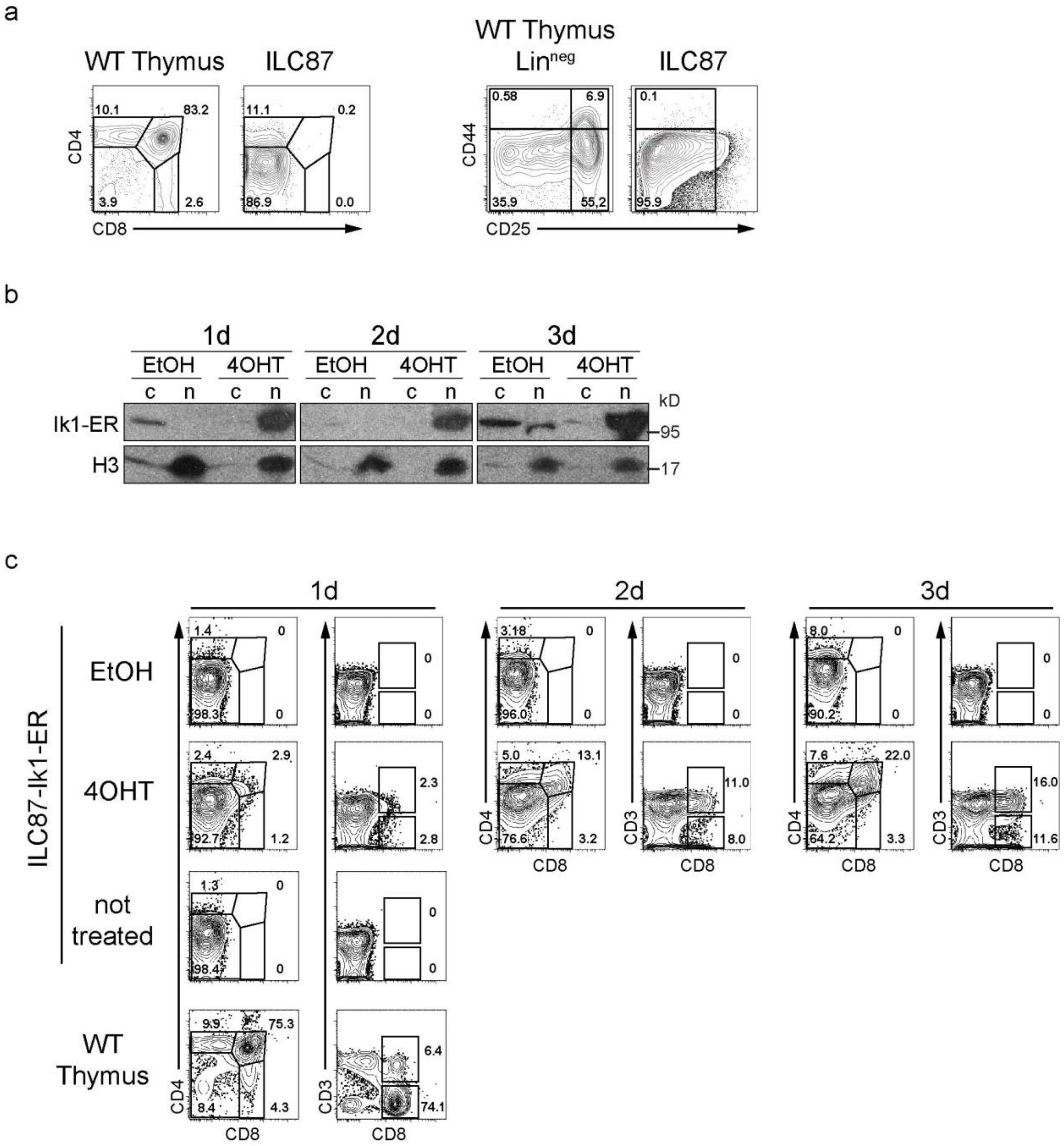

(a) Cell surface expression of CD4, CD8, CD25 and CD44 on ILC87 cells. Staining of WT thymocytes is shown as control. **(b)** Western blot of cytoplasmic (c) and nuclear (n) extracts of ILC87-Ik1-ER cells treated with EtOH or 4OHT for 1-3d as indicated. Histone H3 was used as a loading control. Results are representative of ≥3 independent experiments. **(c)** ILC87-Ik1-ER cells were treated with EtOH or 4OHT for 1-3d, or left untreated as indicated and then analyzed. WT thymocytes were used as reference. Numbers indicate the percentages. Representative of 2 experiments.

Supplementary Figure 8 - Activation of Ikaros in ILC87-Ik1-ER cells induces H3K27 trimethylation.

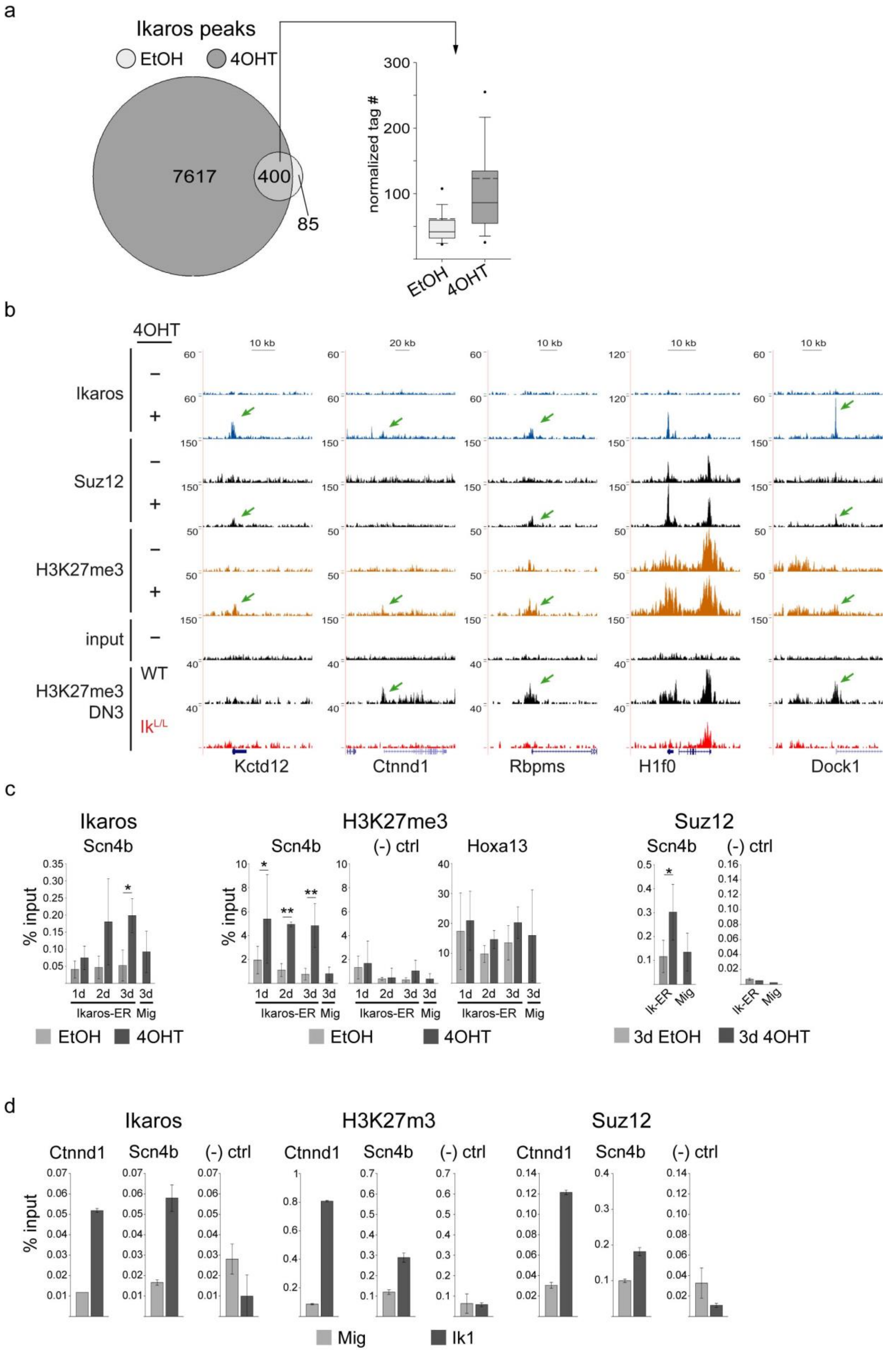

(a) Venn diagram of the Ikaros peaks identified in 4OHT- and EtOH-treated ILC87-Ik1-ER cells by ChIP-seq. The intersection contains the 400 peaks that overlap. Box plots of the normalized tag count distributions of the 400 overlapping Ikaros peaks (right) indicate small Ikaros binding in the EtOH control at these sites which is strongly increased in the 4OHT-treated cells. Dashed lines indicate means, dots indicate the 5th and 95th percentiles. **(b)** Representative genome browser tracks showing ChIP-seq of Ikaros, Suz12 and H3K27me3 in ILC87-Ik1-ER cells treated with 4OHT (+) or EtOH (-) for 1 (Ikaros) or 3 days (Suz12 and H3K27me3). **(c)** ChIP-qPCR analysis of Ikaros, H3K27me3 and Suz12 at the *Scn4b* locus in ILC87-Ik1-ER cells treated with 4OHT or EtOH for the indicated times. Control samples (Mig) are ILC87 cells transduced with the empty MigR1 vector and treated with 4OHT for 3d. Negative control (ctrl) regions were measured with primers annealing to sequences 7.8 kb upstream of the TSS of *Mpzl2* (middle panel) or to exon 6 of *Cd4* (right panel). % input = [(ab ChIP)-(IgG ChIP)]/1% input. Error bars, SD; (*): $p<0.05$, (**): $p<0.01$, two-sample t-test; n=3-7. **(d)** ChIP-qPCR analysis of Ikaros, H3K27me3 and Suz12 at the *Ctnnd1* and *Scn4b* loci in ILC87-Ik1-Bcl2 cells constitutively expressing non-tagged WT Ikaros and ILC87-Mig-Bcl2 control cells. Negative control (ctrl) regions were measured with primers annealing to exon 6 of *Cd4*. % input = [(ab ChIP)-(IgG ChIP)]/1% input. Results are representative of three experiments. Error bars represent the SD of technical duplicates.

Supplementary Figure 9 - Mta2 and Mi2β depletion in ILC87-Ik1-Bcl2 cells.

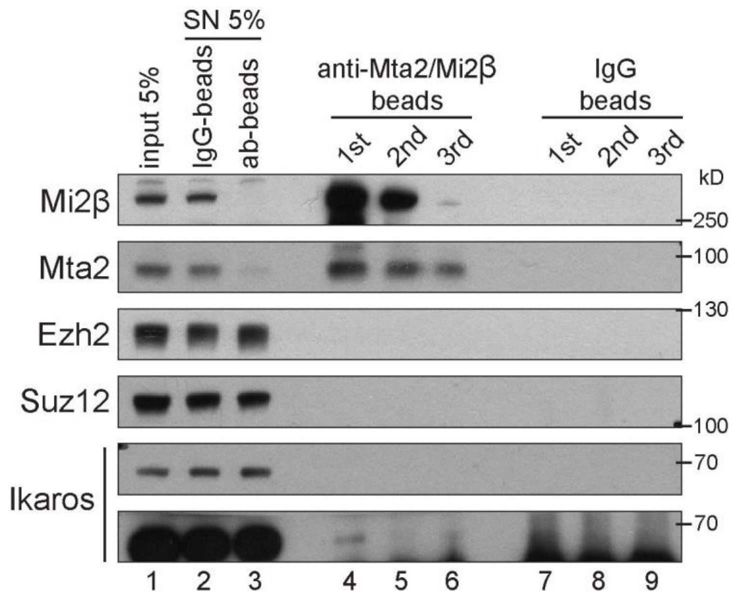

Western blots of Mta2 and Mi2β capture on the beads that were used in the 3 consecutive IP steps for the depletion of these proteins from the nuclear extracts of ILC87-Ik1-Bcl2 cells in Fig. 7g. 1/4 of the Protein A/G beads coupled to anti-Mta2 and anti-Mi2β antibodies (lanes 4-6) or to IgG (lanes 7-9) were analyzed from each IP step (1st-3rd). 5% of the depleted (lane 3) and non-depleted (lane 2) supernatants and 5% input (lane 1) are also shown. An overexposed image of the Ikaros Western blot is shown at the bottom.

Supplementary Figure 10 - Original scans of Western blots in Figures 3, 6 and 7

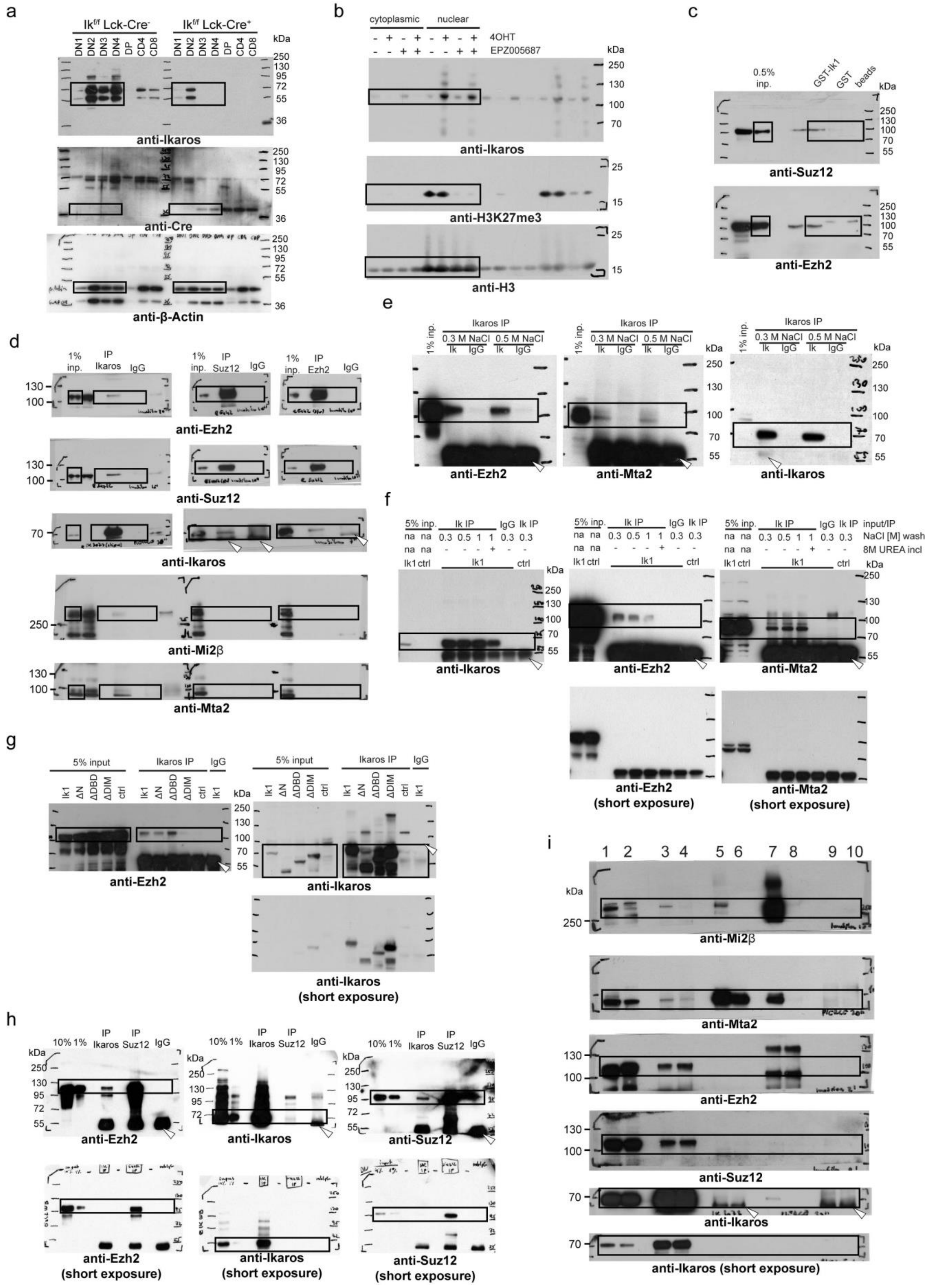

(a) corresponds to Fig. 3c. **(b)** corresponds to Fig. 6c. **(c-i)** panels c-i correspond to Fig. 7 a-g, respectively. Boxed areas correspond to the parts of the scans shown in the figures. White arrowheads point to IgG heavy chains.

Supplementary Figure 11 - Original scans of Western blots in Supplementary Figures 2, 4-7 and 9

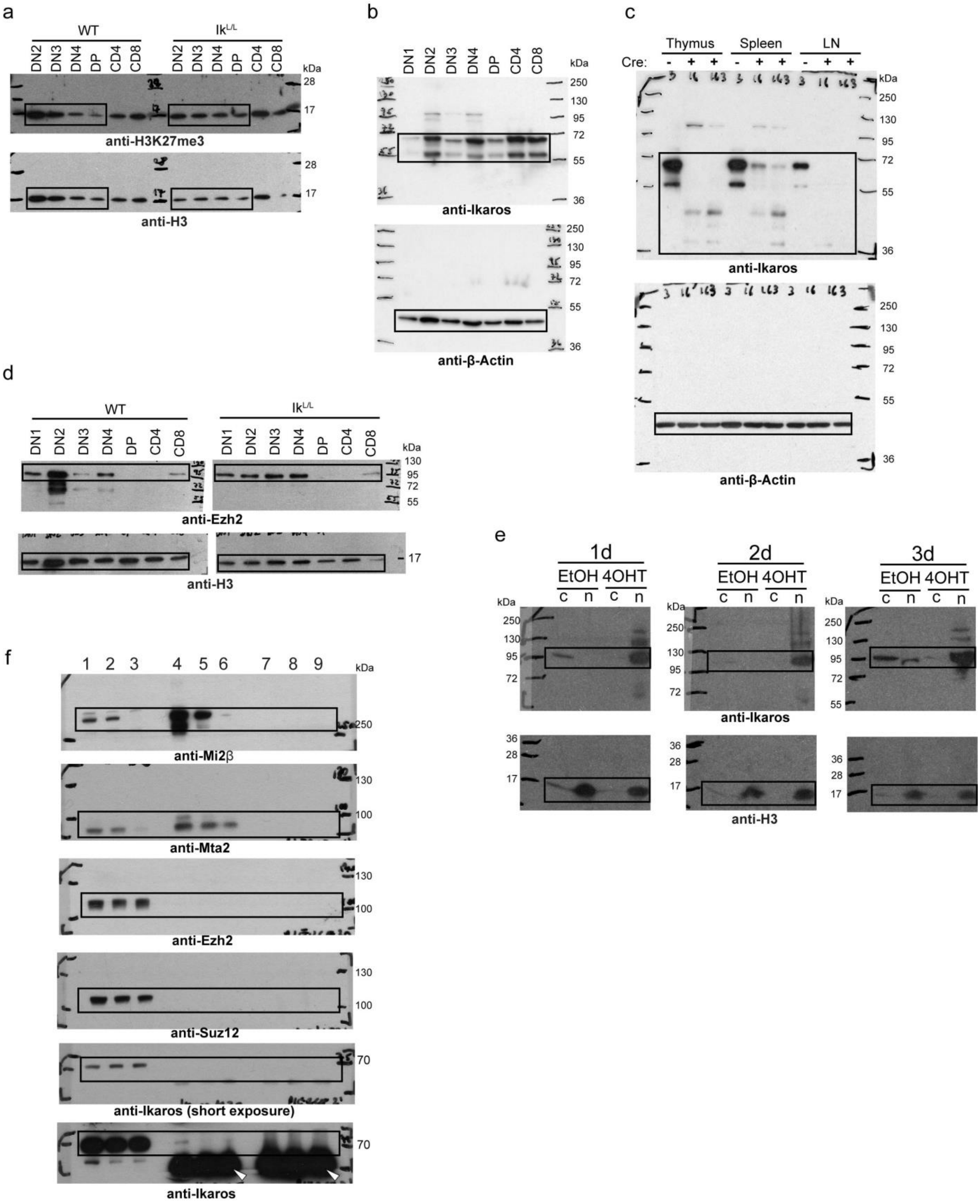

(a) corresponds to Supplementary Fig. 2a. **(b)** corresponds to Supplementary Fig. 4b. **(c)** corresponds to Supplementary Fig. 5c. **(d)** corresponds to Supplementary Fig. 6a. **(e)** corresponds to Supplementary Fig. 7b. **(f)** corresponds to Supplementary Fig. 9. Boxed areas correspond to the parts of the scans shown in the figures. White arrowheads point to IgG heavy chains.

Supplementary Table 1 - 583 genomic regions with >1.8-fold decreased H3K27me3.

Chromosome	Start	End	Gene
chr1	16095800	16097599	Rdh10
chr1	34291400	34296799	Mir5103
chr1	39119800	39121599	Intergenic
chr1	51969800	51975999	Myo1b
chr1	53007800	53010799	1700019D03Rik
chr1	55463200	55464599	Plcl1
chr1	59536600	59544199	Fzd7
chr1	59817800	59825399	Bmpr2
chr1	65230800	65235199	Idh1
chr1	69872600	69873999	Spag16
chr1	78564000	78565599	Intergenic
chr1	82278000	82295999	Irs1
chr1	87830200	87831999	Intergenic
chr1	94719400	94734399	Gpc1
chr1	94742400	94748599	Mir149
chr1	107739800	107742399	Tnfrsf11a
chr1	121358000	121359399	Intergenic
chr1	121538800	121540399	Epb4.1l5
chr1	121542000	121545599	Epb4.1l5
chr1	133856800	133864399	Slc45a3
chr1	133961000	133967799	Mfsd4
chr1	135074200	135080999	Intergenic
chr1	135694400	135697799	Intergenic
chr1	135750800	135752799	Intergenic
chr1	135802000	135807999	Optc
chr1	136192800	136197999	Myog
chr1	136275600	136280199	4933406M09Rik
chr1	137477000	137485599	Nav1
chr1	138692800	138695599	Intergenic
chr1	154469400	154473999	Rgl1
chr1	158239000	158243599	Nphs2
chr1	173364400	173373599	F11r
chr1	174135400	174137199	Pea15a
chr1	174171000	174176999	Atp1a4
chr1	180204600	180208999	Intergenic
chr1	181595800	181602999	Sccpdh
chr1	181777400	181778799	Intergenic
chr1	181883600	181892799	Cdc42bpa
chr1	182723000	182725999	Intergenic

chr1	182823400	182825999	Lefty2
chr1	183849600	183853999	Lbr
chr1	188522000	188532799	Tgfb2
chr1	191547800	191558999	Ptpn14
chr1	192795600	192806599	Vash2
chr1	193914200	193916399	Traf5
chr1	194656800	194664399	1700065J18Rik
chr1	194671400	194691799	Sertad4
chr1	194856000	194866999	Syt14
chr2	21061000	21067199	Intergenic
chr2	25649400	25650999	Gm13539
chr2	27530000	27536199	Rxra
chr2	28301200	28305799	Ppp1r26
chr2	30458600	30463799	Cstad
chr2	30784000	30785599	Intergenic
chr2	31798600	31809599	Aif1l
chr2	32165600	32179399	Mir199b
chr2	32866200	32867799	Slc2a8
chr2	34088800	34114199	Pbx3
chr2	35473200	35480799	Dab2ip
chr2	35585800	35589999	Intergenic
chr2	50995200	51006599	Rnd3
chr2	58415600	58423399	Acvr1
chr2	59446600	59453999	Tanc1
chr2	60976400	60978199	Intergenic
chr2	70495000	70496399	Gorasp2
chr2	71463000	71470199	Intergenic
chr2	73790400	73792399	Intergenic
chr2	75441400	75451999	Intergenic
chr2	75864600	75874999	Ttc30a1
chr2	75946400	75948599	Ttc30a1
chr2	79230000	79231399	Cerkl
chr2	79338800	79340599	Intergenic
chr2	84488000	84491799	Ctnnd1
chr2	90622200	90624999	Agbl2
chr2	91033600	91038399	Nr1h3
chr2	91289000	91304399	Lrp4
chr2	92209400	92213199	Gyltl1b
chr2	92367400	92372599	Intergenic
chr2	92752600	92760799	Syt13
chr2	94281000	94281799	Api5
chr2	101345800	101349199	Intergenic

chr2	101634400	101641599	Prr5l
chr2	102024200	102027999	Ldlrad3
chr2	102274400	102310999	Fjx1
chr2	113237800	113239999	Fmn1
chr2	116947200	116949799	Spred1
chr2	119898000	119904399	Intergenic
chr2	130996200	131000599	Spef1
chr2	131176000	131178399	Rnf24
chr2	132297200	132302399	Intergenic
chr2	145498200	145504399	Intergenic
chr2	152235200	152241799	Zcchc3
chr2	152284000	152285399	Defb23
chr2	152548400	152552199	Intergenic
chr2	152558600	152568399	Id1
chr2	152736600	152740599	Mylk2
chr2	154230600	154236599	Snta1
chr2	155211800	155212999	Trp53inp2
chr2	155339200	155340799	Ggt7
chr2	156895400	156910399	9830001H06Rik
chr2	164610400	164612799	Snx21
chr2	165207800	165215199	Zfp334
chr2	166832000	166833399	Ddx27
chr2	167172400	167177199	B4galt5
chr2	167384000	167388799	Intergenic
chr2	169121600	169124599	Intergenic
chr2	169457000	169462199	Tshz2
chr2	172170400	172173999	2010011I20Rik
chr2	172675800	172678399	Intergenic
chr2	172686400	172689799	Intergenic
chr2	172759000	172767999	Bmp7
chr2	181402400	181413399	Sox18
chr3	9172200	9174799	Intergenic
chr3	9301600	9304199	Intergenic
chr3	27605600	27614599	Fndc3b
chr3	58439800	58443199	Fam194a
chr3	58806800	58814199	Med12l
chr3	68669600	68676999	1110032F04Rik
chr3	80603200	80607999	Gria2
chr3	81876400	81879599	Gucy1b3
chr3	83993200	83996999	Gm6525
chr3	84055600	84065999	Intergenic
chr3	86971000	86984599	Kirrel

chr3	88304600	88309799	Lmna
chr3	90024400	90025399	Rab13
chr3	90317000	90318599	S100a1
chr3	95908800	95909799	Otud7b
chr3	95962400	95969199	Mtmr11
chr3	100906000	100921999	Ptgfrn
chr3	100963600	100970799	Intergenic
chr3	101179800	101207199	Igsf3
chr3	102905000	102907799	Intergenic
chr3	103427200	103429399	Syt6
chr3	104847000	104859599	Cttnbp2nl
chr3	104880000	104881599	4930564D02Rik
chr3	107984400	107991199	Amigo1
chr3	108086200	108090799	Sort1
chr3	114606800	114608199	Olfm3
chr3	116414000	116416599	Slc35a3
chr3	127407800	127410599	Neurog2
chr3	129878400	129887999	Col25a1
chr3	130780400	130791399	Intergenic
chr3	132605600	132615399	Npnt
chr3	142056600	142059199	Pdlim5
chr3	142361200	142366999	Ccbl2
chr3	145769600	145773799	Wdr63
chr3	146066600	146068999	Ssx2ip
chr3	148618000	148624199	Lphn2
chr3	153385800	153388599	St6galnac3
chr4	9593000	9597399	Asph
chr4	48550000	48557399	5730528L13Rik
chr4	53171400	53172599	Abca1
chr4	53453600	53456199	Slc44a1
chr4	57793600	57797199	Intergenic
chr4	62333400	62337399	Rgs3
chr4	86873000	86877799	Slc24a2
chr4	94715800	94718399	Jun
chr4	97986400	97988799	Intergenic
chr4	98784400	98790999	Dock7
chr4	107835600	107842599	Echdc2
chr4	108073600	108081599	Gpx7
chr4	116268600	116271399	C530005A16Rik
chr4	116766400	116771399	Ptch2
chr4	118905600	118907999	Lepre1
chr4	119406400	119408999	Intergenic

chr4	119819000	119824799	Intergenic
chr4	119912200	119915999	Intergenic
chr4	119921600	119964399	Foxo6
chr4	122909000	122918799	Heyl
chr4	129053800	129055999	Zbtb8a
chr4	132309800	132317599	Smpdl3b
chr4	132564000	132567399	Ahdc1
chr4	133958000	133959399	Pafah2
chr4	134318800	134328399	Ldlrap1
chr4	135686400	135692599	Intergenic
chr4	138468600	138469599	Otud3
chr4	141150400	141158999	Fblim1
chr4	141792600	141796599	Kazn
chr4	142886000	142893399	Pdpn
chr4	147309000	147313199	Plod1
chr4	147511200	147514199	Mad2l2
chr4	155408200	155410599	Tnfrsf18
chr4	155559800	155561999	Agrn
chr5	5514000	5515199	Cldn12
chr5	13396800	13400799	Sema3a
chr5	20208600	20210399	Phtf2
chr5	20559400	20561199	Ptpn12
chr5	23179200	23181199	Intergenic
chr5	31246400	31249399	Cgref1
chr5	36098800	36102999	Ablim2
chr5	42096200	42099399	Rab28
chr5	44485800	44494599	Prom1
chr5	52906600	52908799	Intergenic
chr5	66913000	66916999	Apbb2
chr5	76756400	76757399	Pdcl2
chr5	77444000	77446599	1700023E05Rik
chr5	77959400	77962399	Intergenic
chr5	93432400	93437799	Intergenic
chr5	103769400	103770799	Intergenic
chr5	114624000	114626599	Acacb
chr5	114962800	114965199	Intergenic
chr5	114985200	114990199	Intergenic
chr5	116738600	116740199	Ccdc60
chr5	117570400	117572199	Taok3
chr5	122295400	122296399	Fam109a
chr5	124134400	124136199	Clip1
chr5	128111200	128117399	Glt1d1

chr5	135723200	135729399	Fzd9
chr5	137055800	137058599	Intergenic
chr5	137370600	137372799	Intergenic
chr5	137787200	137796199	Ephb4
chr5	151988600	151995999	Stard13
chr5	152032600	152039399	Intergenic
chr6	8725600	8730799	Ica1
chr6	17312200	17314799	Intergenic
chr6	30409800	30415799	Intergenic
chr6	34403600	34407399	Intergenic
chr6	38826400	38829599	Hipk2
chr6	39504400	39512799	Dennd2a
chr6	42641200	42645199	Fam115a
chr6	45009400	45011399	Cntnap2
chr6	48394600	48399999	Sspo
chr6	51559800	51565599	Intergenic
chr6	54273200	54286799	Prr15
chr6	70960600	70968999	Intergenic
chr6	70997600	71000799	Intergenic
chr6	87927600	87933199	H1fx
chr6	89307800	89314199	Plxna1
chr6	100237800	100239999	Rybp
chr6	100670000	100672999	Gxylt2
chr6	112220800	112227599	Lmcd1
chr6	114992000	114993999	Tamm41
chr6	115162000	115164199	Timp4
chr6	116143600	116145199	Tmcc1
chr6	119398400	119404999	Fbxl14
chr6	119491000	119496199	Wnt5b
chr6	119796800	119801399	3110021A11Rik
chr6	122246400	122249999	Intergenic
chr6	122288200	122289399	Phc1
chr6	124704600	124711599	Atn1
chr6	124804200	124810399	Leprel2
chr6	124857800	124863199	Lag3
chr6	124940000	124955599	Pianp
chr6	125262600	125264599	Ltbr
chr6	125328200	125332999	Plekhg6
chr6	125441600	125446999	Cd9
chr6	125551000	125556799	Vwf
chr6	128304000	128306399	Tulp3
chr6	135014600	135017599	Gprc5a

chr6	140370000	140374599	Plekha5
chr6	145690800	145699799	Intergenic
chr7	4114600	4118399	Cdc42ep5
chr7	17202600	17204799	Grlf1
chr7	19945200	19950799	Ppp1r13l
chr7	25854800	25859999	Grik5
chr7	51690200	51704599	Lrrc4b
chr7	52228600	52230999	Cpt1c
chr7	53037400	53056999	Lmtk3
chr7	63096000	63099999	Cyfip1
chr7	75089200	75104399	Igf1r
chr7	78616400	78618399	Intergenic
chr7	86931400	86939199	Intergenic
chr7	86986200	86995199	Anpep
chr7	87078600	87083999	2610034B18Rik
chr7	87169200	87171399	Zfp710
chr7	89076200	89083199	Hdgfrp3
chr7	89318400	89321799	Sh3gl3
chr7	97574400	97576599	Ccdc89
chr7	105258600	105261599	Myo7a
chr7	105504800	105512199	Tsku
chr7	107756600	107758799	Mrpl48
chr7	108041800	108044599	Mir3102
chr7	108981000	108989799	Inppl1
chr7	116813200	116818999	Intergenic
chr7	123448800	123453399	Intergenic
chr7	125735600	125741799	Tmc5
chr7	134779400	134781599	Intergenic
chr7	135559400	135563599	Rgs10
chr7	135665400	135668599	Bag3
chr7	135755200	135756799	Inpp5f
chr7	141860200	141865199	Dock1
chr7	149556000	149559999	Ifitm10
chr7	150235200	150243799	R74862
chr8	13952200	13967799	2610019F03Rik
chr8	28368800	28372799	Eif4ebp1
chr8	32222400	32224199	Rnf122
chr8	32296600	32298999	Fut10
chr8	35035200	35044199	Rbpms
chr8	35792800	35797599	Intergenic
chr8	35866600	35874399	Dusp4
chr8	47235600	47237199	Intergenic

chr8	49073200	49078599	Wwc2
chr8	86715800	86717599	Intergenic
chr8	97412200	97414399	Intergenic
chr8	107676200	107680199	Intergenic
chr8	107872200	107875199	Slc9a5
chr8	109453600	109463999	Sntb2
chr8	118964600	118967199	Intergenic
chr8	119257400	119260199	Cdyl2
chr8	119385800	119389999	Intergenic
chr8	122784800	122790999	Intergenic
chr8	123824400	123845199	Intergenic
chr8	129040400	129042599	Intergenic
chr9	14660400	14668999	Gpr83
chr9	36603400	36605599	Ei24
chr9	37332800	37341799	Esam
chr9	44846200	44853599	Mpzl2
chr9	44938800	44954399	Scn4b
chr9	46037800	46040399	Apoa1
chr9	51083800	51089999	Gm684
chr9	54764600	54766599	Agphd1
chr9	56708400	56715399	Cspg4
chr9	57311000	57318199	Scamp5
chr9	60590200	60592599	Intergenic
chr9	61967400	61969799	Intergenic
chr9	62383400	62385599	Coro2b
chr9	62956400	62959199	Intergenic
chr9	63772600	63777199	Intergenic
chr9	69958000	69960799	Fam81a
chr9	70051400	70060199	Myo1e
chr9	71568200	71575199	Cgnl1
chr9	71615800	71622199	Cgnl1
chr9	72760600	72764799	Pygo1
chr9	75162400	75163399	Gnb5
chr9	84667600	84669199	4930554C24Rik
chr9	92434400	92438799	Plod2
chr9	95714000	95715999	Pls1
chr9	101151200	101154599	Ppp2r3a
chr9	107606800	107616399	Sema3f
chr9	118836000	118837199	Ctdspl
chr10	6601400	6608399	Plekhg1
chr10	7078600	7079799	H60c
chr10	8602400	8608599	Sash1

chr10	11001400	11003399	Fbxo30
chr10	13041800	13046199	Phactr2
chr10	18036200	18042199	Intergenic
chr10	18461600	18465399	D10Bwg1379e
chr10	21711000	21714199	Sgk1
chr10	43196800	43198999	Bend3
chr10	56093800	56101399	Gja1
chr10	59677200	59684199	Chst3
chr10	66464800	66467599	Intergenic
chr10	66586200	66591999	Jmjd1c
chr10	75241000	75246799	Gstt3
chr10	75259600	75262599	Gstt1
chr10	76046200	76047999	Ftcd
chr10	79048000	79048799	Theg
chr10	79239600	79243199	Fstl3
chr10	80691000	80696599	Atcay
chr10	80986000	80988399	Gna15
chr10	84846800	84853599	Btbd11
chr10	90427200	90437399	Apaf1
chr10	93975800	93982399	Tmcc3
chr10	94726600	94729799	Cradd
chr10	94857200	94863399	Intergenic
chr10	94873000	94886399	Socs2
chr10	98378000	98380399	Atp2b1
chr10	110772600	110773999	Intergenic
chr10	110942200	110948799	Phlda1
chr10	116187000	116188399	Intergenic
chr10	120911400	120913399	Rassf3
chr10	126773200	126780199	Gli1
chr10	127101200	127105199	Nab2
chr10	127175600	127177799	Zbtb39
chr10	128024800	128028999	Erbb3
chr11	4955000	4968599	Gas2l1
chr11	20728200	20738999	1110067D22Rik
chr11	22185400	22191599	Ehbp1
chr11	22510000	22511799	Intergenic
chr11	29271000	29276199	Ccdc88a
chr11	32200400	32201799	Hbq1a
chr11	48670600	48673599	Intergenic
chr11	51062200	51064999	Intergenic
chr11	52570800	52591599	Fstl4
chr11	53331600	53335799	Sept8

chr11	53791200	53795599	Slc22a21
chr11	54714800	54718199	Gpx3
chr11	58418800	58420399	Intergenic
chr11	59988600	59991199	Rai1
chr11	64787400	64788599	Elac2
chr11	68451000	68453999	Intergenic
chr11	68501600	68511199	Myh10
chr11	69009600	69012799	Alox8
chr11	69233000	69236799	Kdm6b
chr11	69413000	69431799	Atp1b2
chr11	69434800	69438799	Sat2
chr11	69507600	69509999	Tnfsf12
chr11	70353000	70358399	Pld2
chr11	71547400	71568999	Wscd1
chr11	72389400	72392399	Intergenic
chr11	77999800	78008799	Rab34
chr11	79786000	79788599	Intergenic
chr11	94879400	94883799	Samd14
chr11	96958000	96964999	Tbx21
chr11	96974000	96980599	Tbx21
chr11	97169000	97174199	Mrpl45
chr11	97297800	97304799	Intergenic
chr11	97553800	97564199	Pcgf2
chr11	98206400	98215399	Ppp1r1b
chr11	98418600	98426599	Intergenic
chr11	98459200	98464399	Gm12
chr11	99103800	99106999	Krt222
chr11	100623000	100624599	Hcrt
chr11	101237400	101238999	G6pc
chr11	102087800	102093799	Hdac5
chr11	102290000	102292599	Grn
chr11	102296000	102301399	Grn
chr11	102973000	102974399	Hexim1
chr11	103220600	103226399	Arhgap27
chr11	103893400	103894999	Intergenic
chr11	107647000	107679399	Cacng4
chr11	108780000	108790799	Axin2
chr11	110398600	110399999	Intergenic
chr11	114324400	114331799	Intergenic
chr11	114500200	114501599	Intergenic
chr11	114586600	114590599	Dnaic2
chr11	115231800	115248799	Cdr2l

chr11	115716200	115724799	Intergenic
chr11	116440200	116442399	Ube2o
chr11	116687800	116689999	Mxra7
chr11	116920000	116922399	Intergenic
chr11	119905800	119912999	Aatk
chr11	121247200	121251199	Rab40b
chr12	4844800	4850599	Fkbp1b
chr12	21115600	21120199	Asap2
chr12	76407000	76413999	Rhoj
chr12	77631800	77636799	Mir5135
chr12	85532800	85534199	C130039O16Rik
chr12	86034400	86044599	Tmem90a
chr12	87419000	87427799	Ift43
chr12	101480200	101481799	Intergenic
chr12	104153000	104155399	Intergenic
chr12	107606400	107625599	Intergenic
chr12	107764000	107769599	Intergenic
chr12	108264600	108266799	Intergenic
chr12	109044800	109046999	Intergenic
chr12	109522200	109524599	Ccdc85c
chr12	114038200	114049799	BC022687
chr12	114112000	114118399	Intergenic
chr13	9794200	9796599	Intergenic
chr13	12632000	12637599	Intergenic
chr13	31075200	31081599	Intergenic
chr13	47021800	47026799	Kif13a
chr13	47367600	47369199	Intergenic
chr13	51342200	51343599	Intergenic
chr13	64444600	64445999	Intergenic
chr13	65276800	65278199	Olfr466
chr13	73594800	73596999	Lpcat1
chr13	75842200	75848399	Ell2
chr13	76076600	76084399	Rhobtb3
chr13	89678200	89682199	Hapln1
chr13	94074200	94078199	Homer1
chr13	95052600	95057999	Scamp1
chr13	111587400	111590199	Intergenic
chr13	112889600	112893399	Intergenic
chr14	8831400	8837199	Abhd6
chr14	9047000	9050199	Kctd6
chr14	18491600	18497599	Thrb
chr14	19873800	19875599	Intergenic

chr14	32745000	32748399	Intergenic
chr14	34259000	34259999	Mapk8
chr14	46183400	46186199	Intergenic
chr14	52533800	52535599	Ndrg2
chr14	52738600	52740999	Rpgrip1
chr14	54878400	54880999	Abhd4
chr14	55047200	55053799	Mmp14
chr14	55188200	55198199	Jub
chr14	55672600	55675599	Intergenic
chr14	56956000	56958399	Intergenic
chr14	60505000	60506399	Atp8a2
chr14	65839800	65841199	Fzd3
chr14	70589400	70596399	Sorbs3
chr14	71003600	71005599	Fam160b2
chr14	75038800	75044999	Htr2a
chr14	100875600	100877999	Intergenic
chr14	102004800	102008599	Tbc1d4
chr14	103378000	103385199	Mir5130
chr14	106290600	106300999	Spry2
chr14	119507000	119508399	Uggt2
chr15	6334200	6340999	Dab2
chr15	25551400	25554399	Myo10
chr15	36310200	36312199	Intergenic
chr15	54109600	54110599	Tnfrsf11b
chr15	57520200	57528399	Zhx2
chr15	58043800	58046399	Fbxo32
chr15	58653200	58654799	Tmem65
chr15	78257000	78260199	Kctd17
chr15	78536800	78551399	Elfn2
chr15	79088400	79094199	Slc16a8
chr15	81060600	81071999	Mchr1
chr15	84048400	84049799	Intergenic
chr15	89207200	89210999	Odf3b
chr15	89326400	89334999	Shank3
chr15	91502400	91504799	Lrrk2
chr15	93347800	93351999	Prickle1
chr15	96083800	96085999	Intergenic
chr15	98955400	98958999	Spats2
chr15	99122000	99124999	Prpf40b
chr15	99632400	99635799	Lima1
chr15	100434000	100436199	Intergenic
chr15	102501600	102505999	Mir688

chr16	13443000	13453799	Mir193b
chr16	20693000	20700599	Fam131a
chr16	21974000	21978199	Liph
chr16	31080000	31081199	AI480653
chr16	32641600	32642399	Tnk2
chr16	34093600	34097799	Umps
chr16	35570600	35572999	Sema5b
chr16	37583200	37586399	Hgd
chr16	38481600	38486199	Cd80
chr16	43501600	43506999	LOC626693
chr16	45093600	45094799	Ccdc80
chr16	45408400	45409199	Cd200
chr16	52448400	52455399	Alcam
chr16	62853400	62855999	Pros1
chr16	78930400	78932799	Chodl
chr16	92335200	92337999	Intergenic
chr16	95419600	95421599	Intergenic
chr17	10527200	10530399	Intergenic
chr17	13063800	13066599	Mrgprh
chr17	23721000	23724599	Zfp13
chr17	28825800	28827999	Mapk14
chr17	33941600	33951999	Kank3
chr17	34395400	34407199	H2-Ab1
chr17	34526800	34530399	BC051142
chr17	35956400	35959999	Ier3
chr17	36001600	36005199	Ppp1r18
chr17	37078000	37086399	Rnf39
chr17	46764600	46766999	Ptk7
chr17	48057000	48060999	Foxp4
chr17	48191200	48196199	Intergenic
chr17	56258800	56264999	Lrg1
chr17	56807600	56809799	Ranbp3
chr17	66869200	66870399	Rab12
chr17	83521200	83524999	Intergenic
chr17	87360400	87366999	Rhoq
chr17	87507000	87510399	Socs5
chr17	87871200	87874999	Intergenic
chr18	4634200	4637599	9430020K01Rik
chr18	34999200	35001599	Reep2
chr18	35021000	35026599	Egr1
chr18	35277000	35281399	Ctnna1
chr18	36031600	36035399	Intergenic

chr18	36689800	36692599	Slc4a9
chr18	37962400	37971599	Pcdhgc3
chr18	37972400	37977399	Pcdhgc4
chr18	53576000	53578599	Ppic
chr18	61069800	61073399	Arsi
chr18	61191800	61199399	Cdx1
chr18	61404800	61406199	Pde6a
chr18	61560200	61564199	Ppargc1b
chr18	61797600	61801599	LOC545261
chr18	62064400	62074799	Ablim3
chr18	65952600	65954599	Sec11c
chr18	66615600	66622799	Pmaip1
chr18	67545800	67557999	Tubb6
chr18	69356000	69362199	Intergenic
chr18	73780800	73788199	Intergenic
chr18	77953000	77960999	Intergenic
chr18	82753000	82754599	Mbp
chr19	5473600	5476399	Efemp2
chr19	10941200	10944799	Tmem132a
chr19	23206200	23219799	Klf9
chr19	24245400	24251799	Tjp2
chr19	32459600	32467399	2700046G09Rik
chr19	36203400	36205199	Ankrd1
chr19	38114800	38122399	Myof
chr19	41729400	41754599	Slit1
chr19	42056200	42058599	Ubtd1
chr19	42219400	42224999	Marveld1
chr19	44473800	44485399	Scd1
chr19	45090200	45093999	Lzts2
chr19	46597600	46599399	Intergenic
chr19	47416600	47420399	Neurl1a
chr19	47927600	47930999	Gsto1
chrX	7782200	7785599	Porcn
chrX	20446800	20448399	Timp1
chrX	35533000	35534799	Zbtb33
chrX	70917600	70920999	Slc6a8
chrX	91785400	91788399	Maged1
chrX	92220000	92222399	Spin4
chrX	96081200	96087399	Ophn1
chrX	162674200	162679199	Gpm6b

Supplementary Table 2 - Genes with 4OHT-dependent increase of H3K27me3 in ILC87-Ik1-ER cells.

Gene	Ikaros binding	Suz12 increase	Decreased H3K27me3 in primary thymocytes
Ahcyl2	+	+	-
Aif1l	+	-	+ [a]
Alcam	+	-	+ [a]
Apbb2	+	+	+ [a]
Arhgap26	+	-	+ [b]
Arhgef10	+/-	+	-
B3gnt6	+	+	-
C77080	+	+	+ [b]
Cacnb3	+	+	-
Chd7	+	+	+ [a]
Coro2a	+	+/-	-
Ctdsp2	+	-	-
Ctnnd1	+/-	-	+ [a]
Cuedc1	+	+	-
Cyfip1	+	-	+ [a]
Dock1	+	+	+ [a]
Dusp6	+	+/-	-
Fam163b	+	-	+ [b]
Fndc3b	+	+	+ [a]
Fnip2	+	-	-
Fosl2	+	+/-	-
Gfra2	+	+	-
Gli1	+	+	+ [a]
Gls2	+	+	+ [b]
Gpr83	+	-	+ [a]
H1f0	+	+	+ [a]
Hopx	+	+/-	-
Ier3	+/-	+	+ [a]
Igf1r	+	+	+ [a]
Il17rd	+	+/-	-
Itga11	+	+/-	-
Jun	+	+	+ [a]
Kctd1	+	+	-
Kctd12	+	+	+ [a]
Lhx2	+/-	-	-
Lipg	+/-	-	+ [b]
Lpcat1	+	+	+ [a]
Lzts2	+	+/-	+ [a]
Mgat4b	+	+	+ [b]
Nab2	+	+/-	+ [a]
Nfia	+	-	+ [b]
Nkd1	+	+	-
Parva	+	+	-
Pcgf2	+	+	+ [b]
Ppp1r9b	+	+	-
Pvrl1	+	+	+ [b]
Rbpms	+	+	+ [a]
Rnf182	+	-	+ [b]
Scamp5	+	+/-	+ [a]
Scn4b	+	+	+ [a]
Sertad4	+	+	+ [a]
Shb	+	+	+[b]
Slc45a3	+	+	+ [a]
Smpdl3b	+	-	+ [a]
Tbkbp1	+	+	+ [b]
Tnfsf11	+	+	+ [b]
Trio	+	+	-
Ubtd1	+/-	-	+ [a]
Vash1	+	-	+ [b]
Wasf1	+	+	-
Xylt1	+	+	+ [b]

Genes with increased H3K27me3 and Ikaros binding in 4OHT-treated ILC87-Ik1-ER cells (see Fig. 5a and Supplementary Fig. 8b for specific examples). Genes in italics were identified through visual assessment of the data. The rest were selected bioinformatically as having an Ikaros peak in 4OHT-treated cells which overlapped with a H3K27me3 island that increased >1.8x between EtOH- and 4OHT-treated cells. The second column documents Ikaros binding that coincides with H3K27me3: (+) strong binding; (+/-) small peak but clearly above background. The third column documents increased Suz12 binding after 3d of 4OHT treatment: (+) clear increase; (+/-) small peak close to background level; (-) absent or unchanged Suz12 peak. The last column indicates whether H3K27me3 is decreased at the corresponding regions in primary $Ik^{L/L}$ thymocytes: [a] identified among the 583 regions in Fig. 2b; [b] visual inspection of the data confirmed clear decrease of H3K27me3 at the corresponding region though it did not pass the bioinformatic selection threshold.

Supplementary Table 3- Biological functions associated with genes whose H3K27 trimethylation is regulated by Ikaros.

Biological Process	p-value	Genes
Cell Morphology	$1.41x10^{-6}$	NAB2,MYH10,PLD2,INHA,SLC6A9,ATP2B1,SGK1,CDC42EP2,HLADQB1,RAPH1,AMD1, TSC22D3,CLIP1,SCN4B,TBC1D4,RORA,SEZ6,ARHGEF11,GFAP,WNT5B,GOLPH3,RND 2,OSBPL1A,GJA1,ACP2,LMNA,FLOT1,RHOJ,INHBA,DDR1,OPHN1,DOCK1, RHOQ,CD9,CYFIP1,Dst,IRS1,ALCAM,HDGFRP3,RXRA,RAP2A,MMP14,PTPN14, PTPN12,EIF4EBP1,KLF9,CCDC88A,CSPG4,PDLIM5,IGF1R,PPARGC1B,SORT1, SEMA3F,PEX11A,AKAP5,Pmaip1,IRF4,PTPRK,SH3GL3,DAB2IP,GNAQ,MYOZ1,ERBB3, DOCK7,ID3,GRN,APLP2,MAPK14,PLEKHG5,SPRY2,NFIA,ATP1B2,PEA15,AMIGO1, APBB2,DSCAM,JAK3,Hydin,LRP1,CTNND1
Cellular Assembly and Organization	$1.41x10^{-6}$	MYH10,FHOD1,PLD2,SGK1,ATP2B1,CDC42EP2,RAPH1,CLIP1,SCN4B,SEZ6,Cstad, GFAP,ARHGEF11,HIPK2,GOLPH3,RND2,GJA1,ACP2,LMNA,FLOT1,RHOJ,ARHGEF17,I NHBA,DDR1,SHROOM4,DOCK1,OPHN1,RHOQ,CD9,Dst,CYFIP1,HDGFRP3,RXRA,RAP 2A,CTNNA1,EIF4EBP1,KLF9,CCDC88A,CSPG4,IGF1R,PPARGC1B,SORBS3,SEMA3F,P EX11A,AKAP5,PTPRK,RTKN,DAB2IP,GNAQ,DOCK7,GRN,MAPK14,INPP5J,NFIA,SPRY 2,AMIGO1,APBB2,ALS2CL,DSCAM,Hydin,LRP1,AKAP1,CTNND1
Cellular Development	$1.41x10^{-6}$	NAB2,MYH10,PLD2,INHA,MAGED1,RDH10,SGK1,FNDC3B,RAPH1,TSC22D3,CLIP1,SC N4B,RORA,MCOLN3,SEZ6,GPC1,HIPK2,WNT5B,RND2,AXIN2,GJA1,PDE2A,PPP1R1B, LMNA,FLOT1,RHOJ,MYOF,DDR1,INHBA,DOCK1,OPHN1,RHOQ,CD9, Dst,IRS1,RXRA,SCD,RAP2A,RAB28,MMP14,BMPR2,EIF4EBP1,KLF9,SH3PXD2B,IGF1R ,PPARGC1B,SMOX,CUL9,PEX11A,AKAP5,PTPRK,RTKN,DAB2IP,ACVR1,GNAQ,ERBB3 ,DOCK7,ID3,GRN,MAPK14,PLEKHG5,SPRY2,NFIA,AMIGO1,DSCAM,CTNND1
Cellular Function and Maintenance	$1.41x10^{-6}$	FHOD1,MYH10,PLD2,SGK1,ATP2B1,CDC42EP2,RAPH1,CLIP1,SCN4B,SEZ6,Cstad,AR HGEF11,GFAP,HIPK2,GOLPH3,RND2,GJA1,ACP2,FLOT1,RHOJ,ARHGEF17,MYOF,DD R1,INHBA,SHROOM4,DOCK1,OPHN1,RHOQ,CD9,Dst,CYFIP1,IRS1,HDGFRP3,RAP2A, CTNNA1,EIF4EBP1,KLF9,CCDC88A,IGF1R,PPARGC1B,SORBS3,SORT1,PEX11A,AKA P5,PTPRK,RTKN,DAB2IP,GNAQ,ERBB3,DOCK7,ID3,GRN,MAPK14,INPP5J,SPRY2,NFI A,PEA15,AMIGO1,ALS2CL,DSCAM,JAK3,Hydin,LRP1,AKAP1,CTNND1
Nervous System Development and Function	$1.41x10^{-6}$	NAB2,MYH10,PLD2,MAGED1,SLC6A9,SGK1,ADAM11,RAPH1,SCN4B,RORA,SEZ6,MC OLN3,ZBTB20,CUX2,GFAP,HIPK2,WNT5B,RND2,GJA1,ACP2,PPP1R1B,GRIA2,FLOT1, LMNA,DDR1,DOCK1,OPHN1,RHOQ,CD9,Dst,RAI1,ARHGAP35,HDGFRP3,RAP2A,EIF4 EBP1,FXR2,KLF9,CSPG4,IGF1R,SORT1,SEMA3F,AKAP5,KLC2,PTPRK,SH3GL3,DAB2I P,GNAQ,ERBB3,DOCK7,ID3,GRN,APLP2,PBX3,SPRY2,NFIA,ATP1B2,PEA15,AMIGO1, APBB2,DSCAM
Tissue Development	$1.41x10^{-6}$	NAB2,FHOD1,MYH10,PLD2,SGK1,RDH10,ESRRB,CDC42EP2,RAPH1,CLIP1,SCN4B,SE Z6,MCOLN3,ZBTB20,ARHGEF11,GFAP,GPC1,HIPK2,RND2,AXIN2,GJA1,PDE2A, BCOR,ACP2,LMNA,FLOT1,RHOJ,ARHGEF17,MYOF,DDR1,INHBA,DOCK1, OPHN1,TSHZ1,CD9,Dst,RAI1,IRS1,RXRA,FOXP4,RAP2A,RAB28,MMP14,CTNNA1, BMPR2,PTPN12,EIF4EBP1,KLF9,SH3PXD2B,GF1R,PPARGC1B,SORBS3,LUZP1, AKAP5,PTPRK,RTKN,DAB2IP,ACVR1,GNAQ,ERBB3,DOCK7,ID3,GRN,APLP2,MAPK14, INPP5J,PLEKHG5,NFIA,SPRY2,AMIGO1,APBB2,DSCAM,CHD7,LRP1,AKAP1,CTNND1
Embryonic Development	$6.37\ x10^{-5}$	NAB2,MYH10,RDH10,ESRRB,RAPH1,SCN4B,ARL13B,RORA,MCOLN3,ZBTB20,ARHGE F11,HIPK2,GRSF1,RND2,AXIN2,GJA1,ACP2,BCOR,LMNA,MYOF,INHBA,DDR1, SHROOM4,DOCK1,OPHN1,TSHZ1,CD9,PPP2R3A,RAI1,IRS1,ARHGAP35,PLTP, RXRA,FOXP4,RAP2A,MMP14,CTNNA1,BMPR2,PTPN12,EIF4EBP1,KLF9,CSPG4, IGF1R,PPARGC1B,SORT1,SEMA3F,LUZP1,AKAP5,DAB2IP,ACVR1,GNAQ,ERBB3, ID3,GRN,APLP2,MAPK14,NFIA,SPRY2,ATP1B2,PEA15,APBB2,DSCAM,CHD7,CTNND1
Organ Morphology	$2.05\ x10^{-4}$	NAB2,MYH10,INHA,MMP14,BMPR2,HLADQB1,TSC22D3,PDLIM5,IGF1R,CUX2,PPARG, C1B,GFAP,ARHGEF11,SEMA3F,AKAP5,GJA1,GNAQ,LMNA,MYOZ1,ERBB3,ID3,GPX7,I NHBA,DDR1,TSHZ1,DOCK1,OPHN1,PLEKHG5,IRS1,ATP1B2,NFIA,RAI1,APBB2, ARHGAP35,DSCAM,JAK3,RXRA,CHD7,FOXP4
Reproductive System Development and Function	$2.05\ x10^{-4}$	NAB2,MYH10,INHA,GJA1,SLC6A9,ACP2,PPP1R1B,RDH10,MMP14,GNAQ,ESRRB,ERB B3,DDR1,INHBA,TSC22D3,ASB1,GRN,KLF9,MAPK14,IRS1,IGF1R,DSCAM,RXRA
Lipid Metabolism	$2.23\ x10^{-4}$	SCD,PLD2,INHA,SGK1,BAG3,TSC22D3,IDH1,TBC1D4,RORA,CSPG4,IGF1R,ZBTB20,P PARGC1B,SORT1,LDLRAP1,PEX11A,GOLPH3,DAB2IP,CREB3L3,GNAQ,LMNA,INHBA, CD9,RAI1,IRS1,RBMS1,PLTP,RXRA,LRP1
Molecular Transport	$2.23\ x10^{-4}$	MYH10,SCD,PLD2,INHA,SLC6A9,SGK1,BAG3,TSC22D3,IDH1,TBC1D4,CSPG4,RORA, IGF1R,ZBTB20,PPARGC1B,SORT1,LDLRAP1,PEX11A,GOLPH3,SLC6A8,AKAP5,GJA1, CREB3L3,DAB2IP,GNAQ,LMNA,ERBB3,SLC44A1,INHBA,RHOQ,RAI1,IRS1,PEA15, RBMS1,PLTP,RXRA,LRP1
Small Molecule Biochemistry	$2.23\ x10^{-4}$	SCD,PLD2,INHA,SLC6A9,SGK1,BAG3,AMD1,TSC22D3,IDH1,TBC1D4,CSPG4,RORA, IGF1R,ZBTB20,PPARGC1B,SMOX,SORT1,B4GALT5,GPC1,LDLRAP1,PEX11A,GOLPH3 ,SLC6A8,AKAP5,GJA1,PDE2A,PPP1R1B,CREB3L3,DAB2IP,GNAQ,LMNA,ERBB3, SLC44A1,INHBA,RHOQ,CD9,RAI1,IRS1,PEA15,RBMS1,PLTP,RXRA,LRP1
Cardiovascular System Development	$2.26\ x10^{-4}$	NAB2,MYH10,PLD2,RAB28,RDH10,MMP14,BMPR2,HLADQB1,PTPN12,RORA,CSPG4, PDLIM5,IGF1R,PPARGC1B,ARHGEF11,GPC1,HIPK2,LUZP1,AKAP5,GJA1,PDE2A, BCOR,DAB2IP,ACVR1,GNAQ,LMNA,ERBB3,RHOJ,MYOF,GPX7,ID3,INHBA,DOCK1,

and Function		MAPK14,CD9,PLEKHG5,IRS1,SPRY2,ALCAM,RXRA,CHD7,FOXP4
Organismal Development	2.38 x10^{-4}	NAB2,ST6GAL1,MYH10,INHA,SLC6A9,RDH10,ESRRB,ARL13B,RORA,MCOLN3,CUX2, ZBTB20,ARHGEF11,GPC1,HIPK2,GRSF1,PDE2A,AXIN2,GJA1,ACP2,BCOR,GRIA2, LMNA,RHOJ,MYOF,GPX7,INHBA,DDR1,SHROOM4,OPHN1,DOCK1,TSHZ1,CD9, PPP2R3A,RAI1,IRS1,ALCAM,ARHGAP35,PLTP,RXRA,FOXP4,SCD,RAB28,MMP14, CTNNA1,BMPR2,PTPN1,EIF4EBP1,KLF9,CSPG4,PDLIM5,IGF1,PPARGC1B,SORT1, CUL9,SEMA3F,LUZP1,AKAP5,SH3GL3,DAB2IP,GNAQ,ACVR1,MYOZ1,ERBB3,ID3,GRN ,APLP2,MAPK14,PLEKHG5, NFIA,SPRY2,ATP1B2,PEA15,APBB2,DSCAM,JAK3,CHD7,CTNND1
Cell-To-Cell Signaling and Interaction	2.69 x10^{-4}	ST6GAL1,INHA,ESRRB,,GNB4,RORA,EXOC2,ARHGEF11,TRAF5,HIPK2,WNT5B, GOLPH3,RND2,GJA1,PPP1R1B,GRIA2,P2RX5,LAG3,MYOF,INHBA,OPHN1,DOCK1, RPS6KA6,IRS1,ALCAM,GPR125,RAP2A,MOK,MMP14,CTNNA1,WNT8B,ZBTB33, EIF4EBP1,GSTT1,CSPG4,IGF1R,SORT1,LDLRAP1,PEX11A,AKAP5,KLC2,PTPRK, SH3GL3,RTKN,GNAQ,ACVR1,ERBB3,ID3,MAPK14,PBX3,SPRY2,PEA15,LRP1
Cellular Movement	5.26 x10^{-4}	FHOD1,ST6GAL1,MYH10,PLD2,FNDC3B,AMD1,TSC22D3,RORA,GFAP,ARHGEF11,B4 GALT5,GPC1,HIPK2,WNT5B,GJA1,PDE2A,GRIA2,LAG3,LMNA,FLOT1,INHBA,DDR1, DOCK1,RPS6KA6,CD9,CYFIP1,IRS1,ALCAM,ARHGAP35,PLTP,RXRA,RAP2A,RAB28, MMP14,PTPN14,CTNNA1,BMPR2,PTPN12,CCDC88A,SH3PXD2B,CSPG4,IGF1R,SORB S3,SORT1,LDLRAP1,SEMA3F,PTPRK,ACVR1,ERBB3,ID3,GRN,MAPK14,PLEKHG5,NFI A,SPRY2,APBB2,JAK3,LRP1,CTNND1
Cellular Compromise	5.99 x10^{-4}	MYH10,OSBPL1A,GJA1,SGK1,RTKN,GRIA2,LMNA,ESRRB,GPX7,MAPK14,RHOQ,CSP G4,SGIP1,IGF1R,HIPK2
Respiratory System Development and Function	8.4 x10^{-4}	NAB2,GJA1,SLC6A9,RDH10,MMP14,GNAQ,BMPR2,TSHZ1,MAPK14,CD9,PBX3,RAI1,S PRY2,PPARGC1B,DSCAM,RXRA

Ingenuity Pathway Analysis of the biological functions associated with genes that had decreased H3K27me3 in $Ik^{L/L}$ DN or DP cells (392 genes represented on the Affymetrix 430 2.0 array). The biological functions with the most significant enrichment are shown, as well as the associated genes.

Supplementary Table 4 - PCR primers

ChIP primers

gene		Sequence	Amplicon's approximate mapping position relative to TSS [bp]	Used in
Mpzl2	fw	ACTGCTGTTAGGAGGCCGTAGAGA	-7800	Fig. 1d; Fig. S8c
	rev	TTTGGCCATAGTCACATAGGTTCC		
	fw	GTTTTGATTGAAGTTCCGCACAC	-2500	Fig. 1d
	rev	ACGCCCAGTTCCTTCCAGTTA		
	fw	CAGCTTGCTGTTGTGCCTCTTACC	-100	Fig. 1d
	rev	CTGCCCACAAACCAAAAGCCTG		
	fw	TGTGGAGAGAAAAGGATGCC	-500	Fig. 1d; Fig. 3c
	rev	ACTCAGCAGAAAGAAACCAAG		
	fw	TGGCTTTCCCTGATGTATGG	100	Fig. 1d
	rev	GGCAATGGTAGAAAATCCTAGA		
	fw	AGGGCTTTCTGATCTCAGGT	500	Fig. 1d
	rv	CAGACAAAATCCTCCCACGG		
	fw	CGGTAAGGCTGTGAAGAAAGTGAG	100	Fig. 1d
	rv	CTGCAATGGAGGGAAGGAAGAG		
	fw	ATCTGGACGTTGGGGCTTATGG	4100	Fig. 1d
	rv	CAACCCTACTCACCCCTCATTCAG		
Ctnna1	fw	TAGCCCTATGCTTTCTACACTC	-8000	Fig. 1d
	rv	TACTTCTCTGGAATTGTCTCCTG		
	fw	TAGCAGAAGCCAAACAGAAAGAG	-2200	Fig. 1d
	rv	TGAACATGCTGACAATCCAGAAG		
	fw	GATACAGTGGCAAACAACTTCAC	-1000	Fig. 1d
	rv	TAAACTCCTAATCCTCCAGCAC		
	fw	TGAGGATCCCGTTCTACTGT	-500	Fig. 1d
	rev	ACCCTGTACCCTAGTCCAAG		
	fw	CGGACGGGCGGGAGACAAA	0	Fig. 1d
	rev	CCGAGGGGAGGTACGAAGAGTTA		
	fw	GGTCGGGCAGTGGGGTGTTGTAGG	500	Fig. 1d; Fig. 3c
	rev	CGTGGGCCGGGCAGGAATG		
	fw	TTGACTGGATTCGTAACCTTG	1000	Fig. 1d
	rv	AAATTCCATACACACTCACATC		
	fw	AGTGTAGTTTCTCTCCTGACC	2000	Fig. 1d
	rv	CCTATTCATCCTATTCCTTCATCC		
	fw	CTGACATAACAGGCATGAACCAC	7800	Fig. 1d
	rv	TTCGGCATACACCCAGATACAC		
Ctnnd1	fw	GATTCTGCCTATTGTCCCAG	-600	Fig. 3c
	rev	ATAGTTTGTTAGCCTTTCTTCTC		
	fw	TCAGCTCCCTGATTTATGCC	0	Fig. 1d; Fig. S8d
	rv	TTTCCCCTCAGATCCAACAC		
CD9	fw	GGTGGGAAGAAGGAAGATGG	-200	Fig. 1d; Fig. 3c
	rv	TTCTGGGACTTCGGTTTCAC		
CD3d	fw	TTTTCTGAATACCCATGAGTCTGTT	0	Fig. 1d
	rv	CCTCACGGGTCCATCCTC		
Hoxa13	fw	GATACAATTAGTTGTTCTGATCTTC	-600	Fig. 1d; Fig. 3c; Fig. S8c
	rev	TTGTTTCCTGTTGGTTCCAG		
Scn4b	fw	TGGAGAGGAAGAGGGCTG	-300	Fig. 3c; Fig. 6d; Fig. S8c,d
	rv	CGGAGCTGAAGGGAGGAC		
Fjx1	fw	GTCAAGGCGGCGCTGTAGCA	1400	Fig. 3c
	rv	GAGAGCGGACTGCTAGGCGC		
Ptgfrn	fw	TTCCGGGGCCGGGAGTTCAA	300	Fig. 3c
	rv	CCTCACGTGTGCCGGGGGAG		
Tbx21	fw	CAGGTGGTTGCTGAGCGCGA	600	Fig. 3c
	rv	CGCGGGGCTCTACCCAGGGC		
Zfp334	fw	ATTGTGGCGCACAGAGGCCC	-100	Fig. 3c
	rv	AGCACCCTTTGGAGAACCGT		
Prom1	fw	GCCAGCAACCGCCCACAAGA	1200	Fig. 3c
	rv	CTTCCTGCAGCTACTGCTTC		
Cd4	fw	CACAACGAGAATGGACTAAT	17200 (exon6)	Fig. S8c,d
	rv	TGGGTACTGGAGGACATC		
Tubb2a	fw	GACAGAGGCAAACTGAGCACC	3300	Fig. 3c
	rv	CAACGTCAAGACGGCCGTGTG		

RT-qPCR primers

gene			Used in
Scn4b	fw	CGAGAGAAGAAGAAGGAGTG	Fig. 6b, e
	rv	CAGATAGGACTTGGCATCAG	
Hprt	fw	GTTGGATACAGGCCAGACTTTGTTG	Fig. 6b, e
	rv	GATTCAACTTGCGCTCATCTTAGGC	
